# Supplementary material for: Selective and noncovalent targeting of RAS mutants for inhibition and degradation
Source: Nat Commun. 2021 May 11;12:2656. doi: 10.1038/s41467-021-22969-5 (PMC8113534; doi:10.1038/s41467-021-22969-5)
Supplement: Supplementary file 1 — Supplementary Information [file 41467_2021_22969_MOESM1_ESM.pdf]

Supplementary Information

Selective and Noncovalent Targeting of RAS Mutants for Inhibition and Degradation

Kai Wen Teng[1], Steven T. Tsai[1], Takamitsu Hattori[1], Carmine Fedele[1], Akiko Koide[1,2], Chao Yang[3], Xuben Hou[3], Yingkai Zhang[3], Benjamin G. Neel[1,2], John P O'Bryan[4,5], Shohei Koide[1,6*]

[1]Perlmutter Cancer Center, New York University Langone Medical Center, New York, NY, USA; [2]Department of Medicine, New York University School of Medicine, New York, NY, USA; [3]Department of Chemistry, New York University;
[4]Department of Cell and Molecular Pharmacology, Medical University of South Carolina, Charleston, SC, USA;
[5]Ralph H. Johnson VA Medical Center, Charleston, SC, USA;
[6]Department of Biochemistry and Molecular Pharmacology, New York University School of Medicine, New York, NY, USA;

*Corresponding author. E-mail: Shohei.Koide@nyulangone.org

Supplementary Method 1
Supplementary Tables 1–5
Supplementary Figures 1–12
Supplementary References

Supplementary Method 1. Mass spectrometry analysis of 12VC1 Pull-Downs.

Sample Preparation for Mass Spectrometry. Affinity purification was performed using biotinylated 12VC1 immobilized on Dynabeads M-280 streptavidin beads (three technical triplicates) using 7.2 mg of cell lysates as the input. After the affinity capture step, the beads were resuspended in 100 μL of Tris buffered saline (TBS, 50 mM Tris-Cl pH7.5, 150 mM NaCl) containing 5 mM $MgCl_2$ and 0.2 mM TCEP). Prior to mass spectrometry (MS) analysis the samples were further reduced with 2μL of 0.2M dithiothreitol (Sigma) for one hour at 57°C. Samples were cooled to room temperature followed by alkylation with 2μL of 0.5M iodoacetamide (Sigma) at room temperature in the dark for 45 minutes. Proteins were digested with 500 ng of sequencing grade modified trypsin (Promega) overnight at room temperature with agitation. The solutions were transferred to new tubes and the digestion halted by adding 100 μL of beads slurry consisting of 90 μL 5% formic acid, 0.2% trifluoroacteic acid (TFA), and 10 μL of R2 50 μm POROS beads (Applied Biosystems) to each sample. The tubes were placed on a shaker for 3 hours at 4°C. The beads were loaded onto C18 ziptips (Millipore) that had been equilibrated with 0.1% TFA by centrifugation on a microcentrifuge for 30 seconds at 6,000 rpm. The tubes were rinsed three times with 0.1% TFA and the rinse solution transferred to the corresponding C18 ziptips. The ziptips were washed with 0.5% acetic acid and peptides were eluted with 40% acetonitrile in 0.5% acetic acid followed by 80% acetonitrile in 0.5% acetic acid. Organic solvent was removed using a SpeedVac concentrator. The desalted peptides were reconstituted in 0.5% acetic acid.

Mass Spectrometry Analysis. An aliquot of each sample was loaded onto an Acclaim PepMap trap column (2 cm x 27 μm) in line with an EASY-Spray analytical column (50 cm x 75 μm ID PepMap C18, 2μm bead size) using the auto sampler of an EASY-nLC 1000 HPLC (Thermo Fisher Scientific) with solvent A consisting of 2% acetonitrile in 0.5% acetic acid and solvent B consisting of 80% acetonitrile in 0.5% acetic acid. The peptides were gradient eluted into a Thermo Fisher Scientific Q Exactive mass spectrometer using the following gradient: 5 - 35% in 60 min, 35 - 45% in 10 min, followed by 45 - 100% in 10 min. Full MS spectra were recorded with a resolution of 70,000, an AGC target of 1e6, with a maximum ion time of 120 ms, and a scan range from 400 to 1500 m/z. Following each full MS, MS/MS spectra were acquired using data dependent acquisition on the top 20 ions with a resolution of 17,500, an AGC target of 5e4, maximum ion time of 120 ms, one microscan, 2 m/z isolation window, and Normalized Collision Energy (NCE) of 27.

For the targeted analysis of peptides unique to either G12V or wild type KRAS, another aliquot of each of the triplicate affinity purification samples was loaded onto an Acclaim PepMap trap column (2cm x 27μM) in line with an EASY-Spray analytical column (50 cm x 75 μm ID PepMap C18, 2 μm bead size) using the auto sampler of an EASY-nLC 1200 HPLC (Thermo Fisher Scientific) with solvent A consisting of 2% acetonitrile in 0.5% acetic acid and solvent B consisting of 80% acetonitrile in 0.5% acetic acid. The peptides were gradient eluted into a Thermo Fisher Scientific Q Exactive HF-X mass spectrometer using the following gradient: 5 - 26% in 60 min, 26 - 40% in 10 min, followed by 40 - 100% in 10 min. Full MS spectra were recorded with a resolution of 45,000, an AGC target of 1e6, with a maximum ion time of 45 ms, and a scan range from 400 to 1500 m/z. Following each full MS, MS/MS spectra were acquired only on the +2 charge state of the following peptides: KRAS(G12V)-specific peptide LVVVGAVGVGK (499.3239 m/z), KRAS(WT)-specific peptide LVVVGAGGVGK (478.3004 m/z) and common peptides VEDAFYTLVR (606.8166 m/z; shared by KRAS4A, HRAS and NRAS)

and QGVDDAFYTLVR (692.3488 m/z; shared by KRAS4B (WT and mutants)). The targeted MS/MS scans were acquired using a resolution of 60,000, an AGC target of 2e5, maximum ion time of 120ms, one microscan, 1.4 m/z isolation window, and Normalized Collision Energy (NCE) of 27.

Data Processing. The MS/MS spectra were searched against the Uniprot *homo sapiens* reference database (downloaded 02/2019) additionally containing common contaminant proteins and the mutant KRAS4B(G12V) using Sequest HT within Proteome Discoverer 1.4.0.288. The search parameters were: precursor mass tolerance ±10 ppm, fragment mass tolerance ±0.02 Da, trypsin, cleaving C-terminally to lysines and arginines, allowing two missed cleavages, fixed modification of carbamidomethyl on cysteine, variable modification of oxidation on methionine, and variable modification of deamidation on glutamine and asparagine and a 1% peptide and protein FDR searched against a decoy database. The results were filtered to only include proteins identified by at least two unique peptides with high confidence.

The targeted analysis data was searched using Byonic with the following parameters: variable modifications of oxidation on methionine, deamidation on asparagine and glutamine, and a fixed modification of carbamidomethyl on cysteine, fragment mass tolerance of ± 0.02 Da, precursor tolerance of ±20 ppm, trypsin allowing two missed cleavages and a 1% FDR using a standard target-decoy approach. In addition, the targeted spectra were verified by manual inspection.

Supplementary Table 1. Monobodies used in this work and their sequences.

Clone	*K_{Dapp} (nM)	Amino acid sequence
		10 • 20 • βC • 40 • CD βD 50 • 60 • 70 • FG 90 •
12VC1	5.7±2.1	VSSVPTKLEVVAATPTSLLISWDAPAVTVFFYVITYGETGHGVGAFQAFKVPGSKSTATISGLKPGVDYTITVYARGYSKQGPYKPSPISINYRT
12VC3	2.9±1.1	VSSVPTKLEVVAATPTSLLISWDAPAVTVFFYIIAYGETGHGVGAFQAFRVPGSKSTATISGLKPGVDYTITVYARGYSKQGPYKPSPISINYRT
12VC1.1	3.7±0.7	VSSVPTELEVVAATPTSLLISWDAPAVTVFFYVITYGETGHGVGAFQAFKVPGSRSTATISGLEPGVDYTITVYARGYSKQGPYKPSPISINYRT
12VC1.2	24.6±7.1	VSSVPTELEVVAATPTSLLISWDAPAVTVFFYVITYGETGHGVGAFQAFAVPGSRSTATISGLEPGVDYTITVYARGYSKQGPYKPSPISINYRT

*K_{Dapp} is the apparent K_D determined by yeast display binding titration against KRAS(G12C). Amino acid residues highlighted in red are those that are mutated compared with the parental clone 12VC1.

Supplementary Table 2. Kinetic and equilibrium parameters for 12VC1 binding determined from BLI measurements

Target	Nucleotide State	K_D (nM)	S.D.	k_{on} ($M\ s^{-1}$)	S.D.	k_{off} (s^{-1})	S.D.
KRAS(G12C)	GTPγS	24.7	1.0	1.1E+05	2.2E+04	2.7E-03	5.3E-04
KRAS(G12V)	GTPγS	101	39	7.2E+04	8.3E+03	7.0E-03	1.9E-03
KRAS(G12A)	GTPγS	718	99	NA	NA	NA	NA
KRAS(G12S)	GTPγS	686	57	NA	NA	NA	NA
KRAS(WT)	GTPγS	9600	800	NA	NA	NA	NA
KRAS(G12C)	GDP	ND	ND	ND	ND	ND	ND
KRAS(G12V)	GDP	ND	ND	ND	ND	ND	ND
KRAS(G12A)	GDP	ND	ND	ND	ND	ND	ND
KRAS(G12S)	GDP	ND	ND	ND	ND	ND	ND
KRAS(WT)	GDP	ND	ND	ND	ND	ND	ND

NA, parameter not available, because data were analyzed in the equilibrium mode.
ND, parameter not determined, because binding signals were too low for curve fitting.
K_D and standard deviations (SD) are from three technical replicates.

Supplementary Table 3. Data collection and refinement statistics.

PDB accession number	HRAS(G12C)/12VC1 (7L0G)	HRAS(WT)/12VC3 (7L0F)
**Data Collection**		
Space group	P1 21 1	P1 21 1
Cell Dimensions		
a,b,c (Å)	71.83, 62.60, 123,40	73.01, 64.82, 127.12
α,β,γ (°)	90, 101.22, 90	90, 102.37, 90
Resolution (Å)	50.00-2.54(2.58-2.54)	50.00-1.98(2.01-1.98)
R_{merge}	0.152(0.501)	0.115(0.767)
$I/\sigma I$	8.62(3.21)	14.35(2.78)
Completeness(%)	98.9(99.9)	96.0(95.3)
Redundancy	4.9(5.2)	5.0(4.9)
**Refinement**		
Resolution (Å)	41.87-2.54	44.84-1.98
No. reflections	35,157	77,312
R_{work} / R_{free}	0.199/0.228	0.159/0.191
No. atoms		
Protein	8084	8102
Ligand/ion	132	132
Water	53	641
B-factors		
Protein	55.38	35.55
Ligand/ion	41.63	23.98
Water	40.01	39.13
R.m.s deviations		
Bond lengths (Å)	0.01	0.02
Bond angles (°)	1.27	1.85
**Interface analysis**		
Interface area (Å^2)[a]		
HRAS	853	861
Monobody	794	829

Number of crystals used per data set: 1. Highest resolution shell is shown in parenthesis. Interface analysis was performed by PISA[1]. [a]The solvent-accessible surface area of the indicated molecule that is occluded by its binding partner.

Supplementary Table 4. A list of primers used in this study and their sequences.

Primer	Sequence
T7	5’-TAATACGACTCACTATAGGG-3’
FN5'-1_D3S	5’-GATCCGTTTCTTCTGTTCCGACCAAACTGGAAGTTGTTGCTGCG-3’
FN5'-2_D3S	5’- CGTTTCTTCTGTTCCGACCAAACTGGAAGTTGTTGCTGCG-3’
FN3'1	5’-GTTACTAGGTACGGTAGTTAATCGAG-3’
FN3'-2	5’-TCGAGTTACTAGGTACGGTAGTTAATCGAG-3’
KRas_G12_rev	5’-CACTCTTGCCTACGCCACCAGCTCCAACTACCAC-3’
KRas_G12C_rev	5’-CACTCTTGCCTACGCCACAAGCTCCAACTACCAC-3’
KRas_G12D_rev	5’-CACTCTTGCCTACGCCATCAGCTCCAACTACCAC-3’
KRas_G13D_rev	5’-CACTCTTGCCTACGTCACCAGCTCCAACTACCAC-3’
KRas_addKEKMSKDG_rev	5’-GTGGTGGTGCTCGAGTCAACCATCTTTGCTCATCTTTTCTTTATGTTTTCGAATTTCTCGAAC-3’
NheI_mcherrF	5’-ATGAAGGCTAGCATGGTGAGCAAGGGCG-3’
mb_ApAI_R	5’-TTAAACGGGCCCTCAGGTACGGTAGTTAATCGAG-3’
TetOne_ForSeq	5’-GCTGATTTTTTGAGTAAACTTCAAT-3’
TetOne_RevSeq	5’-CCTAAGACAGGAGGGCCGTC-3’
Infusion_EcoRI_FP_F	5’-CCTCGTAAAGAATTCATGGTGAGCAAGGGCGAG-3’
Flag_Mb_F	5’-CTCGAGACTACAAAGACGATGACGACAAGGGCTCCGTTTCTTCTGTTCCGACCAAAC-3’
mcher_flagR	5’-CGTCTTTGTAGTCTCGAGATCTGAGTCCGGACTTGTACAGCTCGTCCATGC-3’
Infus_MB_BAM_R	5’-GAGGTGGTCTGGATCCTTAGGTACGGTAGTTAATCGAGATTG-3’
Infus_EcoRI_HA_VHL_F	5’-CCTCGTAAAGAATTCATGTATCCGTATGATGTTCCGGATTATGCAATGCCCCGGAGGG-3’
VHL_SSSSG_R	5’-ACCTGATGATGATGAATCTCCCATCCGTTGATGTG-3’
SSSSG_MB_F	5’-CATCATCATCAGGTGTTTCTTCTGTTCCGACCAAAC-3’

Supplementary Table 5. Crystal structures used for comparative analysis.

PDB accession code	Description	Reference
5B30	HRAS(WT) GppNHP State 1	Matsumoto et. al. 2016[2]
4EFL	HRAS(WT) GppNHP State 1	Muraoka et. al. 2012[3]
4G0N	HRAS(WT) GppNHP bound to RBD-RAF1	Fetics et. al. 2015[4]
4L9W	HRAS(G12C) GMPPNP	Ostrem et. al. 2013[5]
5B2Z	HRAS(WT) GppNHP State 2	Matsumoto et. al.2016[2]
1LF0	HRAS(A59G) GppNHP	Hall et. al. 2002[6]
1LFD	HRAS(WT) GppNHP bound to RBD-RALGDS	Huang et. al. 1998[7]
1P2S	HRAS(WT) GppNHP	Buhrman et. al. 2003[8]
1XCM	HRAS(G60A) GppNHP	Ford et. al. unpublished
1ZW6	HRAS(Q61G) GppNHP	Ford et. al. 2006[9]
2C5L	HRAS(WT) GTP bound to RBD-PLCε	Bunney et. al. 2006[10]
2RGA	HRAS(Q61I) GppNHP	Buhrman et. al. 2007[11]
2VH5	HRAS(G12V) GTP bound to anti RAS FV	Tanaka et. al. 2008[12]
3DDC	HRAS(WT) GppNHP bound to NORE1A	Stieglitz et. al. 2008[13]
3KKN	HRAS(T35S) GppNHP	Shima et. al. 2010[14]
3L8Y	HRAS(WT) GppNHP bound to cyclen	Rosnizeck et. al. 2010[15]
3L8Z	HRAS(WT) GppNHP	Rosnizeck et. al. 2010[15]
3OIU	HRAS(Q61L) GppNHP	Buhrman et. al. 2011[8,16]
3RRY	HRAS(WT) GppNHP	Buhrman et. al. 2011[16,17]
3TGP	HRAS(WT) GppNHP	Fraser et. al. 2011[18]
4DLR	HRAS(WT) GppNHP	Holzapfel et. al. 2012[19]
4DLT	HRAS(WT) GppNHP	Holzapfel et. al. 2012[19]
4DLU	HRAS(WT) GppNHP	Holzapfel et. al. 2012[19]
4DLV	HRAS(WT) GppNHP	Holzapfel et. al. 2012[19]
4EFM	HRAS(G12V) GppNHP State 1	Muraoka et. al. 2012[3]
4EFN	HRAS(Q61L) GppNHP State 1	Muraoka et. al. 2012[3]
4G3X	HRAS(Q61L) GppNHP bound to RBD-RAF1	Fetics et. al. 2015[4]
4K81	HRAS(WT) GTP bound to RA and PH domains of GRB14	Qamra et. al. 2013[20]
5WDO	HRAS(WT) GppNHP	Bandaru et. al. 2017[21]
5WDP	HRAS(L120A) GppNHP	Bandaru et. al. 2017[21]
5WDQ	HRAS(L120A) GppNHP	Bandaru et. al. 2017[21]
5X9S	HRAS(WT) GppNHP	Ke et. al. 2017[22]
6AMB	HRAS(WT) GppNHP bound to Afadin	Smith et. al. 2017[23]
6CUO	HRAS(WT) GppNHP bound to SOS1 and a small molecule activator	Abbott et. al. 2018[24]
6D5W	HRAS(WT) GppNHP bound to SOS1 and a small molecule activator	Abbott et. al. 2018[24]
6Q21	HRAS(WT)	Milburn et. al. 1990[25]

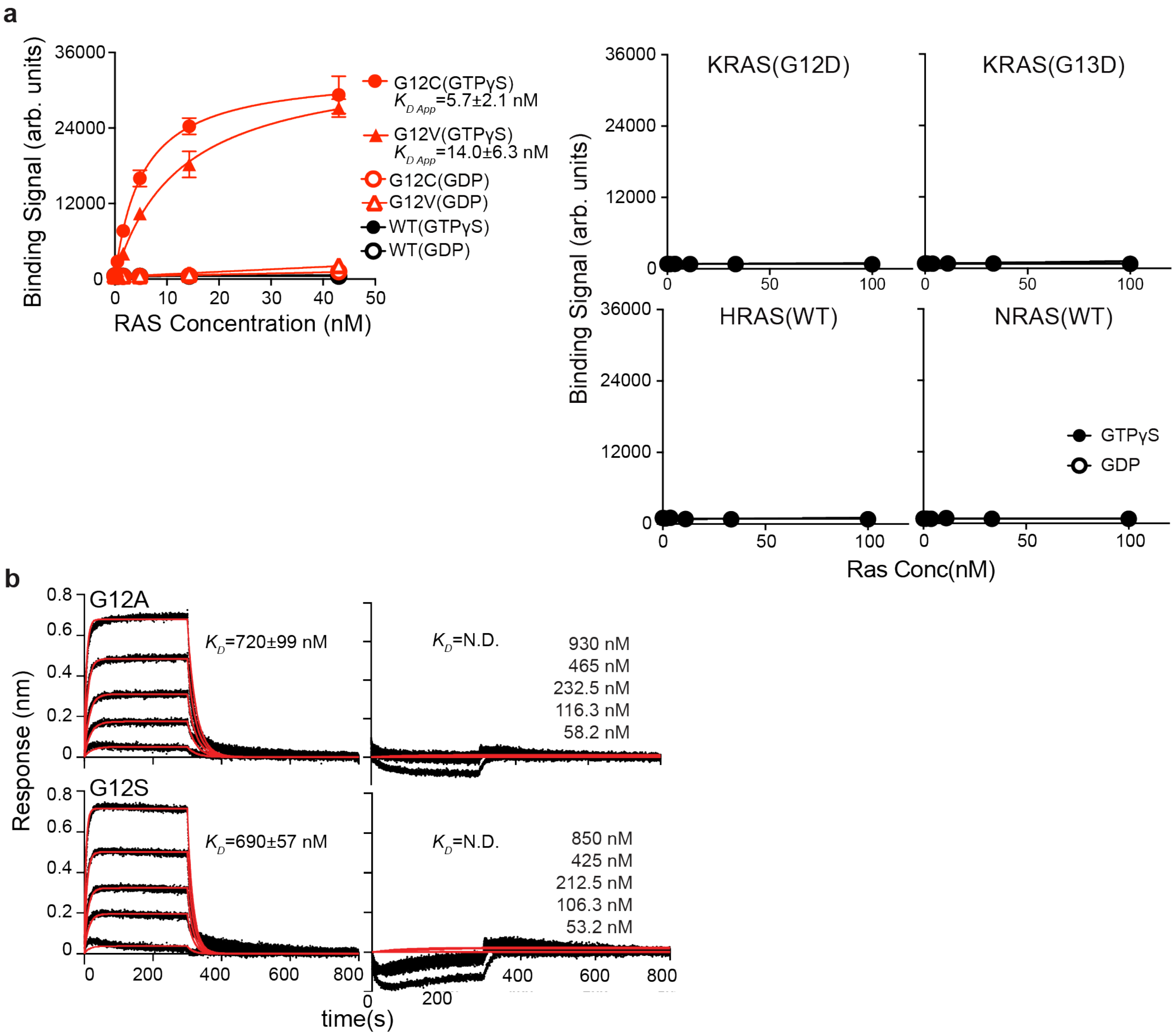

Supplementary Figure 1. Binding of 12VC1 to RAS mutants and isoforms in the yeast display format. **a,** Binding titration of 12VC1 displayed on yeast cells against purified RAS mutants. Binding of 12VC1 in a yeast display format against biotinylated KRAS(G12C), (G12V), and (WT) in either GTPγS or GDP-bound form (top). Binding signals in arbitrary units (arb. units) are the median fluorescence intensity for the 75-95th percentile of the binding-positive population. The apparent K_D values ($K_{D\ App}$) shown are calculated by fitting the data points to the 1:1 binding model. Binding of GTPγS-bound WT and GDP-bound G12C, G12V, and WT were too weak to yield meaningful $K_{D\ App}$. Errors in apparent K_D and plotted data points are the standard deviation (SD), n = 3, technical replicates. Titration of KRAS(G12D), KRAS(G13D), HRAS(WT) and NRAS(WT) in GTPγS and GDP bound nucleotide states showed no binding. **b**, BLI sensorgrams of 12VC1 binding to KRAS(G12A) and KRAS(G12S) in the GTPγS and GDP bound states. Steady state global analysis was performed to calculate the K_D. The K_D values shown are the mean ± SD from n = 3, technical replicates. N.D., not determined due to too weak binding.

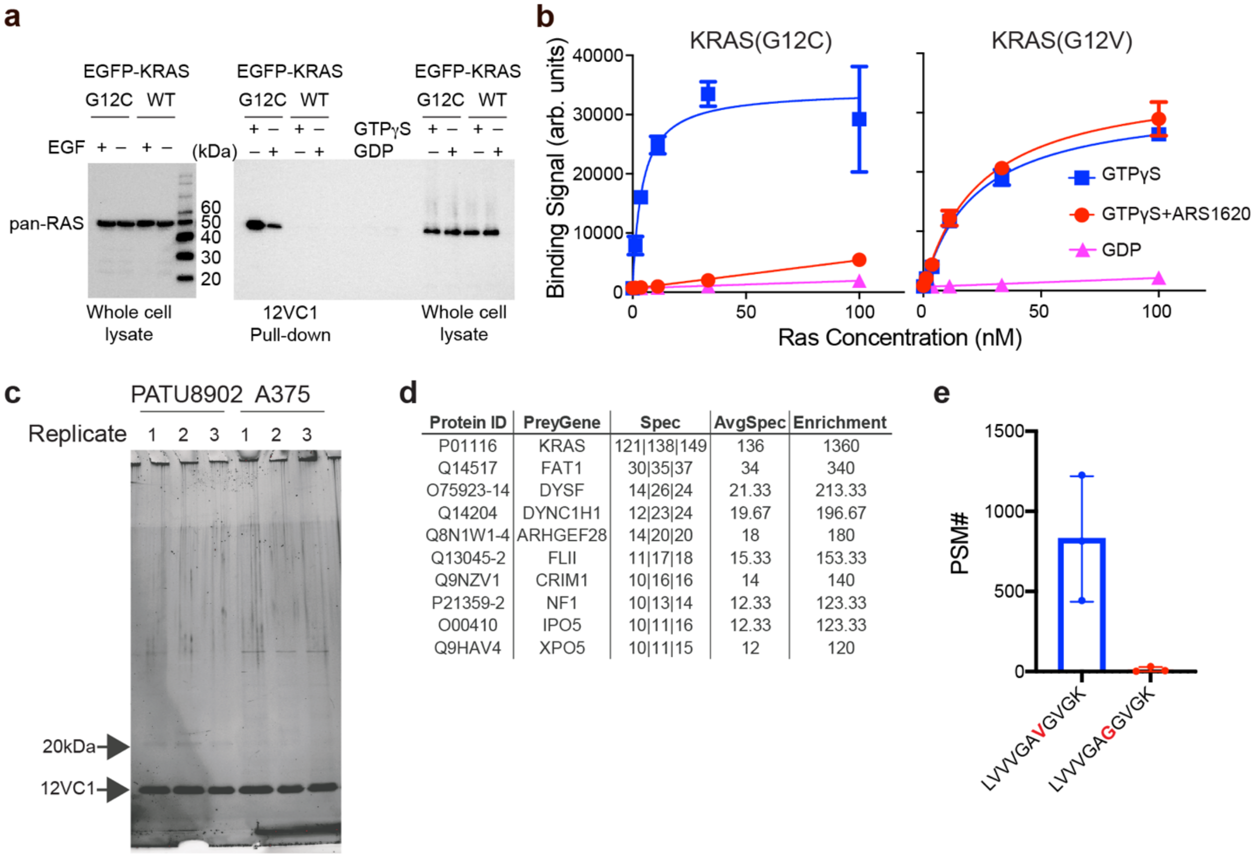

Protein ID	PreyGene	Spec	AvgSpec	Enrichment
P01116	KRAS	121|138|149	136	1360
Q14517	FAT1	30|35|37	34	340
O75923-14	DYSF	14|26|24	21.33	213.33
Q14204	DYNC1H1	12|23|24	19.67	196.67
Q8N1W1-4	ARHGEF28	14|20|20	18	180
Q13045-2	FLII	11|17|18	15.33	153.33
Q9NZV1	CRIM1	10|16|16	14	140
P21359-2	NF1	10|13|14	12.33	123.33
O00410	IPO5	10|11|16	12.33	123.33
Q9HAV4	XPO5	10|11|15	12	120

Supplementary Figure 2. 12VC1 is selective to KRAS mutants in the active state. **a**, Nucleotide and mutant specificity of 12VC1 tested in a pull-down assay (right blot). HEK293T cells were transiently transfected with a plasmid encoding EGFP-KRAS(G12C) or EGFP-KRAS(WT), and lysed after incubation overnight. Lysates (500 µg) were subjected to nucleotide exchange by adding EDTA, followed with an excess concentration of the specified nucleotide. Biotinylated 12VC1 bound to Dynabeads M-280 streptavidin magnetic beads were used to pull down RAS from the lysates. Captured proteins and the inputs were visualized by immunoblotting with an anti-RAS antibody to determine the abundance of captured EGFP-KRAS. A technical replicate was performed with an anti-GFP antibody as the detection reagent with similar results. The identities of the bands were confirmed using a separate blot (left). **b**, Binding of 12VC1 to purified KRAS(G12C) and (G12V) with and without covalent inhibitor ARS1620 tested in the yeast display format. ARS1620 was added to purified KRAS(G12C) and (G12V) during the nucleotide exchange reaction. Binding titration of 12VC1 displayed on yeast was performed. Note that the reaction with ARS1620 abolished the binding of 12VC1 to KRAS(G12C) but not to KRAS(G12V), as expected for the selectivity of ARS1620 to KRAS(G12C). The average binding signal of triplicate measurements and error (standard deviation (SD)) were plotted. **c**, Pull-down experiments were performed with lysates of PATU8902 that contained endogenous KRAS(G12V) and lysates of A375 cells that contained RAS(WT) using 12VC1 as a bait. SDS-PAGE of affinity-purified samples stained with the Krypton staining reagent. **d,** List of proteins that were unique in the lysate of PATU8902 as identified using LC/MS/MS based proteomics (technical replicates, n = 3). The identities and spectral counts of the top 10 most abundant proteins that were uniquely captured from the lysate of PATU8902 are listed. The enrichment score is defined as the ratio of spectral counts recorded from PATU8902 lysate over the spectral counts recorded from the control (A375)

lysate for the listed protein. A value of 0.1 was added to the spectral counts of uniquely identified proteins to avoid divide by 0. **e**, Spectral counts of a KRAS(G12V)-specific peptide, LVVVGAVGVGK, and that of the counterpart specific to wild-type RAS, LVVVGAGGVGK, in pull-down samples of PATU8902, demonstrating that the captured RAS is overwhelmingly KRAS(G12V). The bar center shows average of the three measurements, and the error bar shows SD.

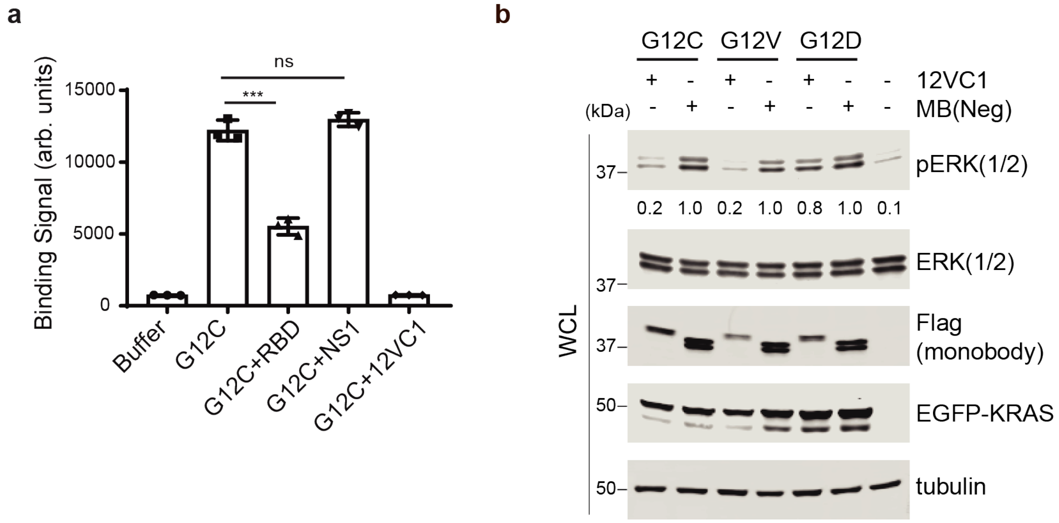

Supplementary Figure 3. Inhibition of Mutant RAS signaling by 12VC1. **a,** Competitive binding assay of 12VC1 against known interaction partners of RAS. KRAS(G12C) (20 nM) was preincubated with excess concentrations (3.875 μM) of purified RAF1-RBD, monobody NS1 or monobody 12VC1, and then their binding to 12VC1 displayed on the yeast surface was measured. Median fluorescence intensity (MFI) and standard deviation are plotted (*n* = 3). KRAS(G12C) preincubated with RAF1-RBD showed significant decrease in binding signal, which suggested that it competed with 12VC1 for binding (*n* = 3, one-way ANOVA, p<0.001). No significant decrease in binding signal was observed when RAS was preincubated with NS1 that binds to the α4-β6-α5 interface away from the switch regions (technical replicates, *n* = 3, one-way ANOVA, p=0.21).**b**, Inhibition of ERK activation in HEK293T cells co-expressing monobody and RAS mutants. HEK293T cells were transiently co-transfected with plasmids encoding EGFP fused to the full length KRAS mutants and mCherry fused to flag-tagged monobodies, 12VC1 or MB(Neg). MB(Neg) is a non-binding monobody control. The EGFP-KRAS mutants were overexpressed compared to the expression level of endogenous KRAS. Whole cell lysates (WCL) were probed for the levels of phospho-ERK (pERK), total ERK (ERK1/2), EGFP-RAS and monobodies (Flag). Representative data from 3 biological replicates are shown.

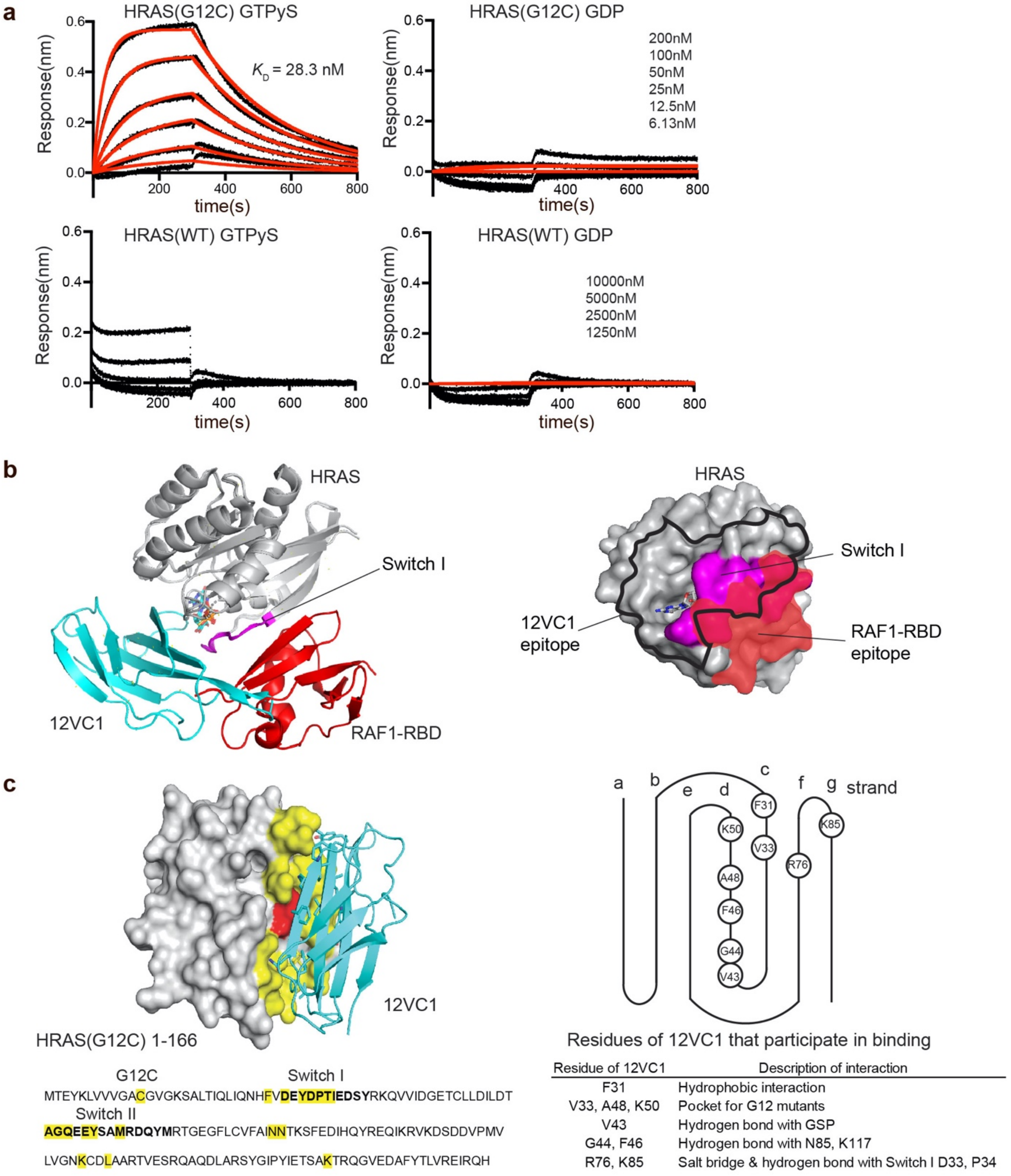

Supplementary Figure 4. The crystal structure of HRAS(G12C) bound to 12VC1. **a**, Selectivity and affinity of 12VC1 to HRAS constructs used for crystallization. BLI binding kinetic measurements of 12VC1 against HRAS(G12C) 1-166 and HRAS(WT) 1-166 in the GTPγS and GDP bound states are shown. BLI data were fitted globally (red). The K_D values of 12VC1 against HRAS(G12C) were 28.3 and 35.9 nM when measured in duplicate. Binding of 12VC1 to GDP-bound HRAS(G12C) and HRAS(WT) were too weak to derive K_D values. **b**, An overlay of 12VC1 and RAF1-RBD (PDB: 4g0n) bound to HRAS. The two structures were superposed using HRAS. The epitopes of 12VC1 (black outline) and RAF1-RBD (red) overlap and include Switch I region (magenta). **c**, Analysis of interacting residues between HRAS(G12C) and

12VC1. 12VC1 is shown in cartoon representation and HRAS(G12C) in surface representation (top). Residues of HRAS(G12C) that are within 4 Å of 12VC1 are highlighted in yellow in the graphics as well as in the sequence. Residue C12 of HRAS(G12C) is highlighted in red in the graphics. Residues in the Switch I and II regions are marked in bold. The right panel shows the positions of residues of 12VC1 that interact with RAS in a schematic representation of the β-strand topology.

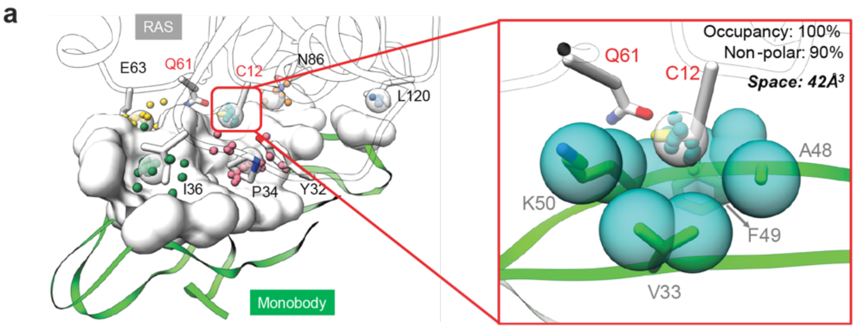

RAS mutant	MD length	Avg. Distance[a]	Avg. RMSD[b]	Loop_RMSD[c]	Loop_dist[d]	Complex stability	Experimental
G12C	250ns	5.1±0.4	3.2±0.6	2.0±0.6	3.1±0.5	Stable	Binding
G12V	250ns	5.3±0.4	2.3±0.2	1.7±0.5	3.0±0.3	Stable	Binding
G12WT	250ns	8.4±1.3	6.8±2.2	4.4±1.6	5.4±1.7	Unstable	No binding
G12D	250ns	7.2±1.5	7.7±3.4	5.5±2.8	5.9±2.6	Unstable	No binding
G13D	250ns	9.0±1.0	7.9±2.4	5.3±1.7	6.5±1.9	Unstable	No binding
Q61L	250ns	9.5±1.1	11.7±2.1	7.1±1.8	8.2±1.8	Unstable	No binding

[a] Average distance between #12 residue in RAS and V33/A48/K50 in monobody
[b] Average RMSD of monobody during MD simulation (complex aligned based on RAS)
[c] Average RMSD of Loop(G44/A45/F46) of monobody during MD simulation (complex aligned based on RAS)
[d] Average distance between K117 (Ras) and G44 (Monobody)

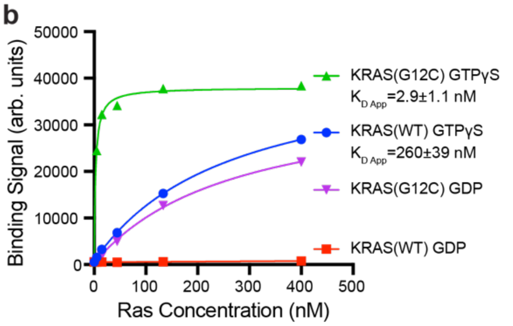

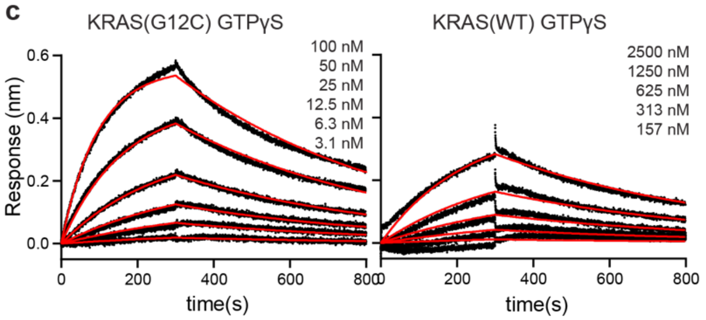

	nuc. state	avg. K_D ± S.D. (nM)
KRAS(WT)	GTPγS	1700 ± 700
KRAS(G12C)	GTPγS	21.0 ± 1.6

Supplementary Figure 5. A surface pocket of 12VC1 provides selectivity to mutant. **a**, A pocket analysis of RAS-monobody complex using *AlphaSpace*[26]. We detected a series of hot-spot binding pockets in the concave surface of monobody 12VC1. The top-scored pocket, which is occupied by Cys12 of RAS, is highlighted. Lower panel: Results of molecular dynamics simulations (MD) of monobody complexed with different RAS mutants. MD revealed that RAS(G12C) and RAS(G12V) formed stable complexes with 12VC1, whereas 12VC1 complexes with the wild type and with the G12D, G13D and Q61L mutants were unstable, which are consistent with experimental results. **b,** 12VC3, a mutant of 12VC1 (containing V33I, T35A and K50R) that binds to RAS(WT) with higher affinity than 12VC1. Yeast display binding titrations of 12VC3 against KRAS(WT) and KRAS(G12C) in the GTPγS and GDP bound states. The uncertainties shown represent errors from nonlinear least-squares fitting of a 1:1 binding model. **c**, BLI sensorgrams of 12VC3 bound to KRAS(G12C) and KRAS(WT). The K_D values are 21 ± 2 nM for KRAS(G12C) and 1777 ± 748 nM for KRAS(WT) (technical replicates, n = 3). Errors represent the standard deviation.

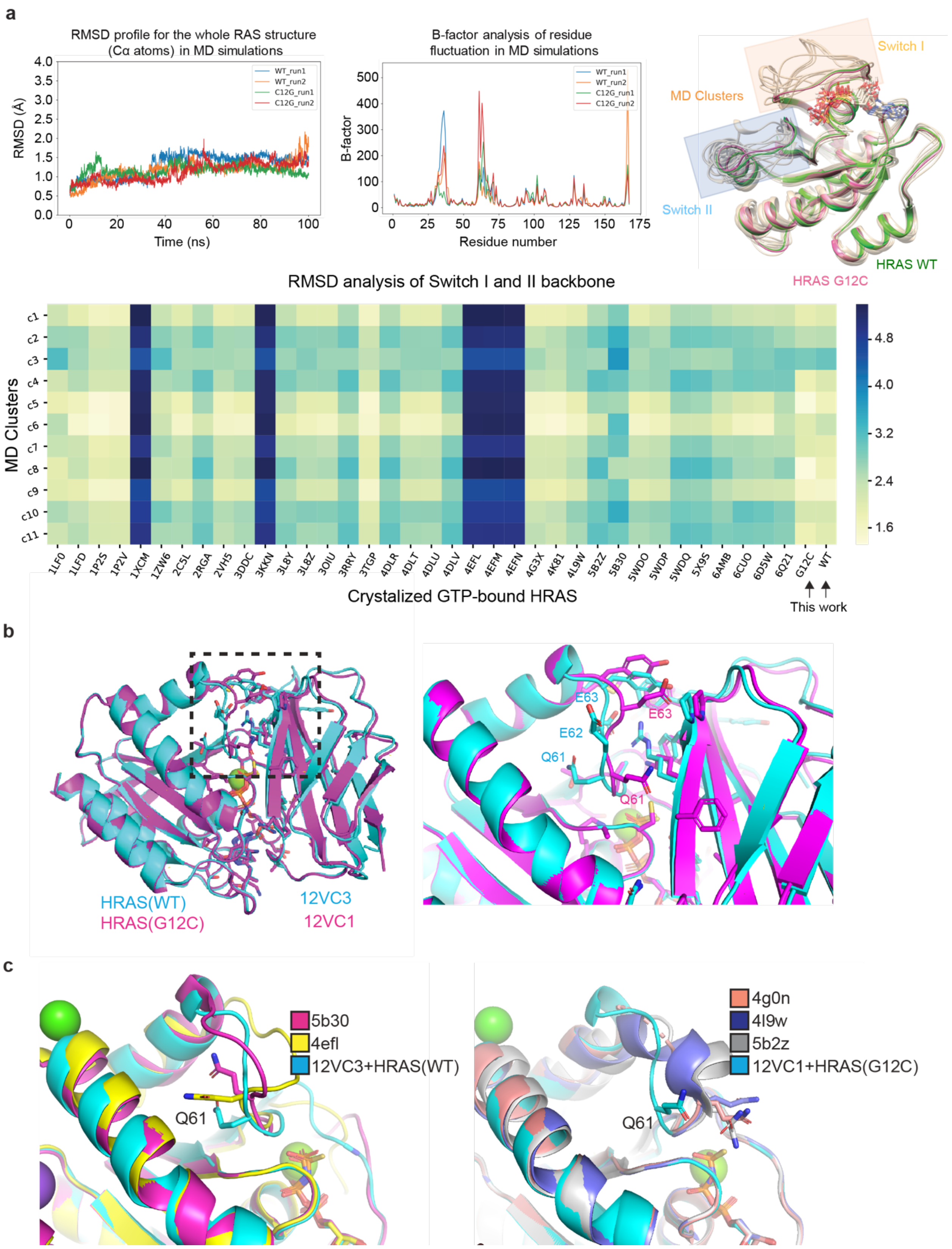

Supplementary Figure 6. Analyses of the crystal structures of 12VC1 HRAS(G12C) and 12VC3 HRAS(WT). **a,** The RMSD analysis (for the Cα atoms of the entire molecule) of HRAS(G12C) and (WT) over 100 ns molecular dynamics simulations after removing the bound

monobodies from the respective complex structures. The results show that these two HRAS conformations were relatively stable and no drastic structural changes occurred (top-left panel). The B-factor analysis of the MD trajectories showing that the most dynamic regions in these two RAS structures were Switch I and Switch II (top-middle panel). Classification of MD trajectories based on the backbone RMSD value of the switch I and II conformations to previously reported crystal structures of RAS bound to GTP analogues (bottom). The HRAS(G12C) and (WT) structures captured by monobodies 12VC1 and 12VC3, respectively, exhibit the same "similarity profile", which suggests that the captured HRAS structures have similar backbone conformation (top-right panel). **b,** The crystal structures of 12VC1-bound HRAS(G12C) (PDB: 7L0G) and 12VC3-bound HRAS(WT) (PDB: 7L0F) were aligned and displayed in ribbon representation. The side chains of residues in the Switch II region of HRAS(G12C) (magenta) and HRAS(WT) (teal) showing that Q61, E62, and E63 are in distinct orientations in the two structures. **c,** Comparing the orientation of Q61 in 12VC3 and 12VC1 bound HRAS(WT) and HRAS(G12C) respectively with other crystal structures of HRAS. 12VC3 bound to a state where Q61 is pointed towards the interior of RAS (left panel). This side chain conformation is found in the structures of GTP-bound RAS in the state 1 conformation[2,3]. Q61 in 12VC1 bound HRAS(G12C) (right panel) is in the state 2 conformation[2]. State 1 and 2 are both observed in HRAS(WT) structures, therefore the free energy difference for the transition between these two states is probably small and is not likely to be a major contributor to the high mutant selectivity of 12VC1.

a

Cell Line	RAS Mutation	Tissue	# of mut. Alleles	KRAS sensitivity (ATARiS score)*
H358	KRAS(G12C)	Lung	1	-1.045
H23	KRAS(G12C)	Lung	1	0.035
PATU8902	KRAS(G12V)	Pancreas	1	-3.092
H441	KRAS(G12V)	Lung	1	-3.092
HPAF-II	KRAS(G12D)	Pancreas	1	N/A
A375	BRAF(V600E)	Skin	N/A	0.135

*a greater negative score indicates higher KRAS dependency

b

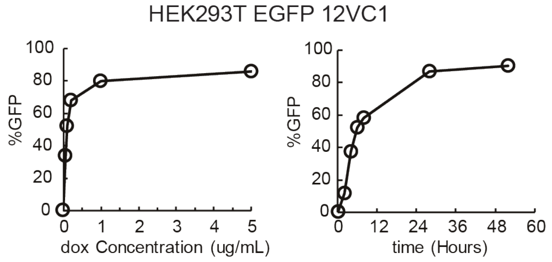

c

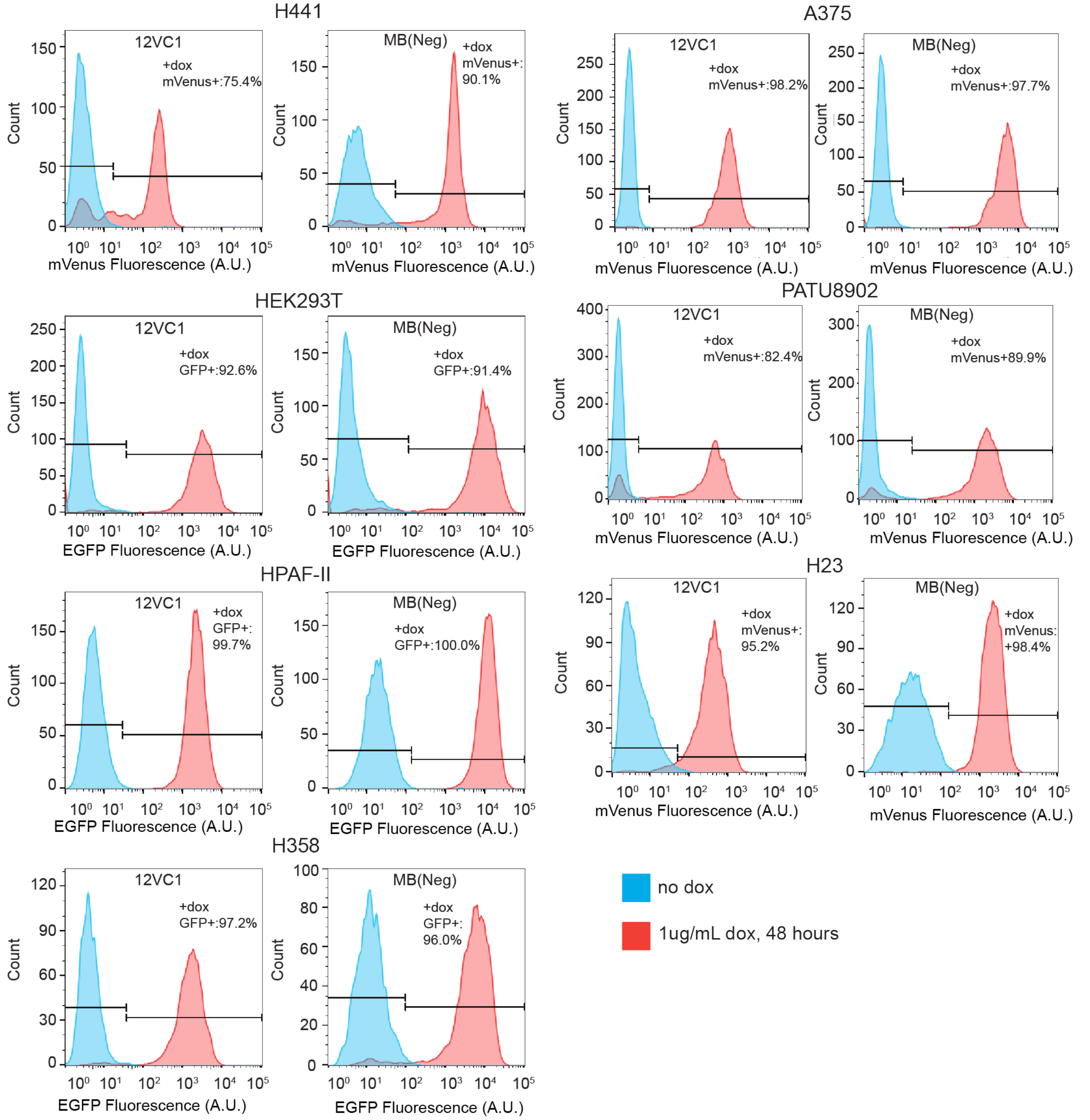

Supplementary Figure 7. Generation of a panel of dox-inducible monobody-expressing cell lines. **a**, Cell lines used for generating dox-inducible stable cell lines. ATARiS scores are obtained from the Project DRIVE online database[27]. **b,** Percentage of HEK293T cells expressing EGFP fused 12VC1 as a function of dox concentration (24 hr. induction) or time (with 1 μg/mL dox). In the presence of 1μg/mL dox, induction for 48 hours was enough to reach the maximal induction of monobody. **c,** Expression profiles of FP-flag-monobody in the generated cell lines.

The parental cell lines are indicated above the graphs. In some cell lines mVenus were used instead of EGFP to reduce the toxicity associated with high expression levels of EGFP. Both mVenus and EGFP were detected through the GFP channel on the flow cytometer. Without dox in the media there was very low basal expression of the monobodies (blue). After 48 hours of induction with 1µg/mL dox, greater than 75% of the cell population became GFP positive (red) for all cell lines.

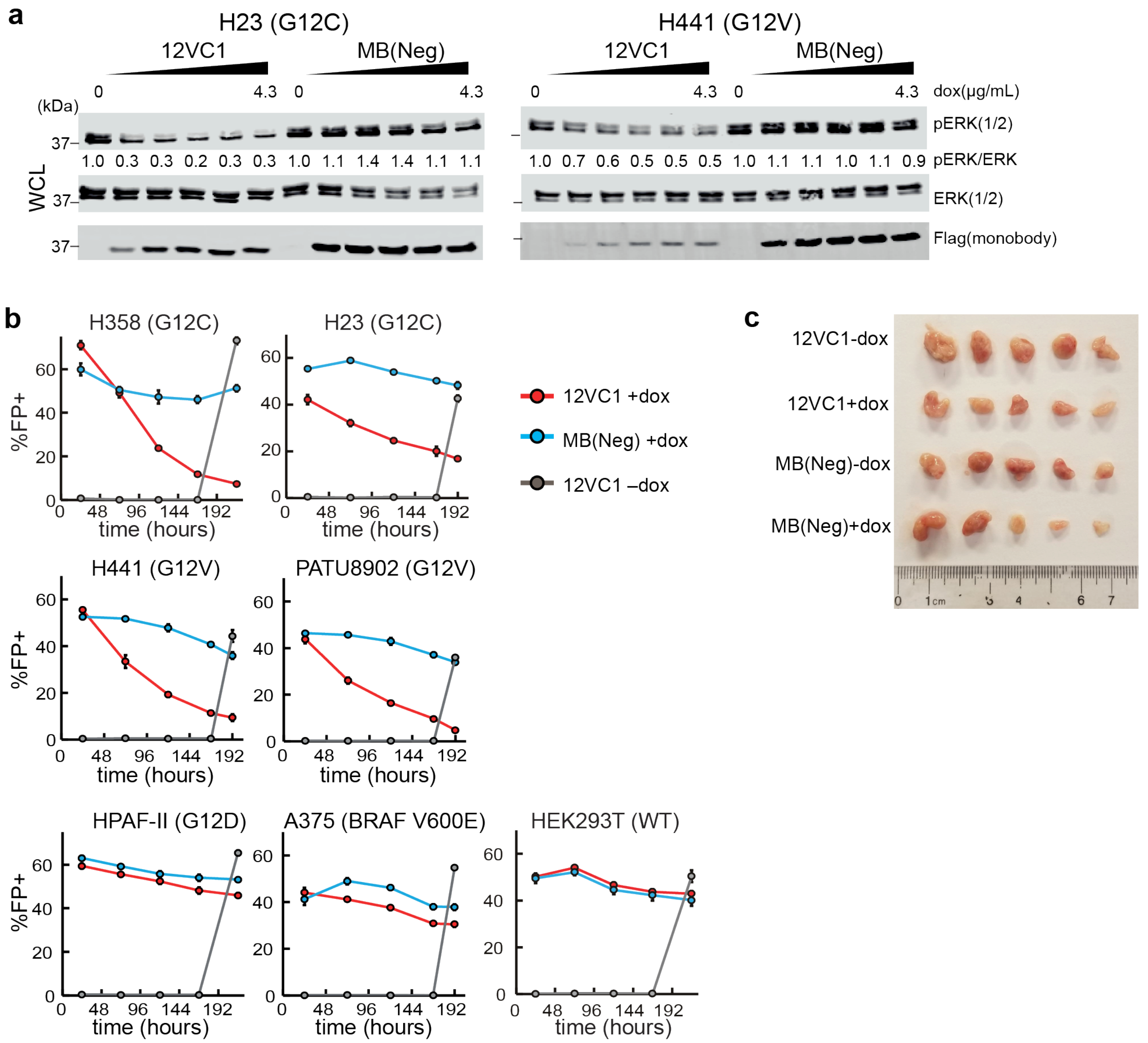

Supplementary Figure 8. Effects of 12VC1 expression on mutant RAS-driven cancer cells and solid tumor. **a,** Effects of 12VC1 on ERK activation in additional cancer cell lines related to Figure 3. Signaling experiments have been reproduced with less samples with similar results. **b,** Effects of 12VC1 expression on proliferation determined using mixed cultures, related to Figure 3b. Dox-inducible cell lines expressing fluorescent protein-fused 12VC1 (red) or a nonbinding control monobody, MB(Neg), (blue) were mixed with respective parental cells at an approximately 1:1 ratio. The mixed cultures were grown in the presence of 1 μg/mL dox for the duration of the entire experiments (red and blue) or just 24 hours before the last time point (gray), a control that eliminates the possibility of growth inhibition due to viral transduction. The fractions of the fluorescence positive (FP+) cell populations, representing the monobody-expression cells, were monitored over time using a flow cytometer. Growth inhibition results in a reduction of the FP+ population. Average of the measurements were plotted. Error bars represents s.d. (biological replicates, *n* = 3). For most data points, error bars are within the size of the symbols. **c**, Tumors extracted from PATU8902 xenografts at the end of the experiment, related to Figure 3c. Tumors were much smaller across the board for 12VC1+dox compared with 12VC1-dox. Tumors are sorted from the largest to the smallest. Dox appeared to have

minimal effect on tumor growth for a majority of the PATU8902 tumors expressing MB(Neg). Expression of the monobody MB(Neg) was not detected in the smallest tumor of the MB(Neg)+dox group, indicating that the tumor failed to engraft in this particular mouse. Quantification of tumor weights is shown in Fig. 3c.

a

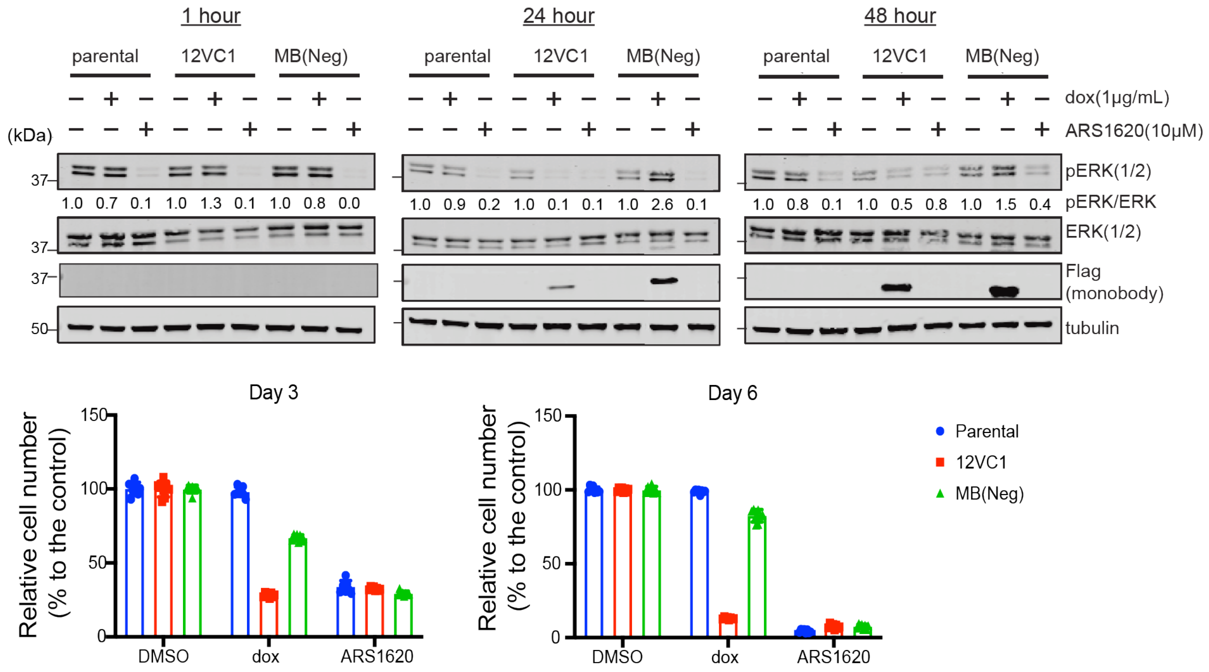

b

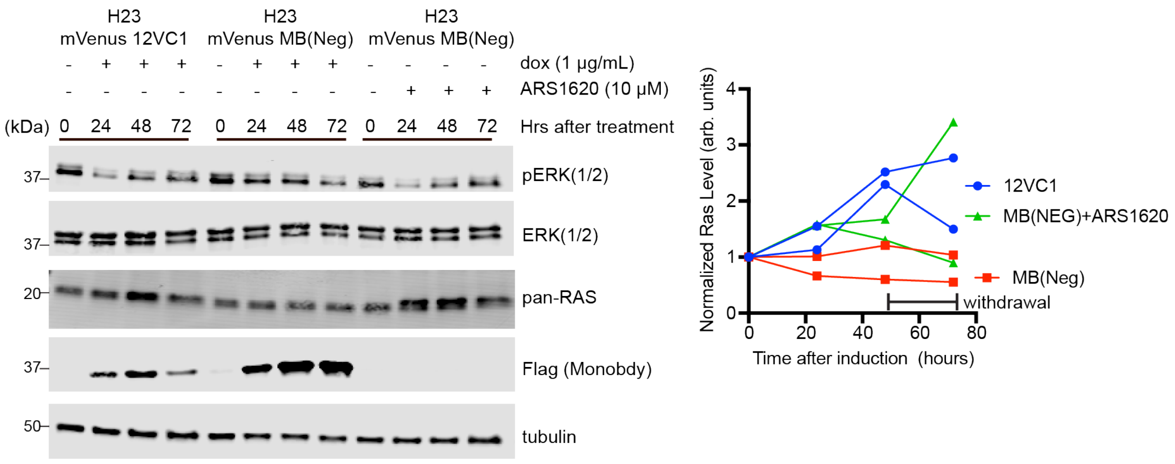

Supplementary Figure 9. Comparisons of a covalent inhibitor (ARS-1620) with the noncovalent monobody inhibitor 12VC1 in the H358 and H23 cell lines. **a**, ERK activation and viability of the H358 cells under dox-induced monobody expression or in the presence of the covalent RAS inhibitor ARS1620. 12VC1 expression and ARS1620 treatment each effectively inhibited ERK activation in the H358 cells after 24 and 48 hours. Both inhibitors led to similar decreases in viability after 3 and 6 days, respectively (bottom graphs). The mean and s.d. (biological replicates, *n* = 8) are shown. **b,** The H23 cells expressing the mVenus-flag-12VC1 fusion or mVenus-flag-MB(Neg) under a dox-inducible promoter were treated with either doxycycline or ARS1620 to examine the effects of covalent versus non-covalent inhibition on the amount of total RAS (representative data shown, biological replicates, *n* = 2, both replicates are plotted). An increase in the total RAS amount was observed after 48 hours under both covalent and non-covalent inhibition, demonstrating that this increase is not exclusively observed under the inhibition with 12VC1. An increase in pERK levels was observed during the

period from 24 to 48 hours after inhibition, which correlated with the increase in total RAS level. By contrast, expressing MB(Neg) did not impact the level of RAS in cells, as expected. After 48 hours, media containing inhibitors and dox were replaced with serum-free and dox-free media to remove ARS1620 and suppress 12VC1 production. However, the RAS level stayed elevated 24 hours after the removal of the inhibitors, revealing a long-term effect of direct RAS inhibition on the total RAS level.

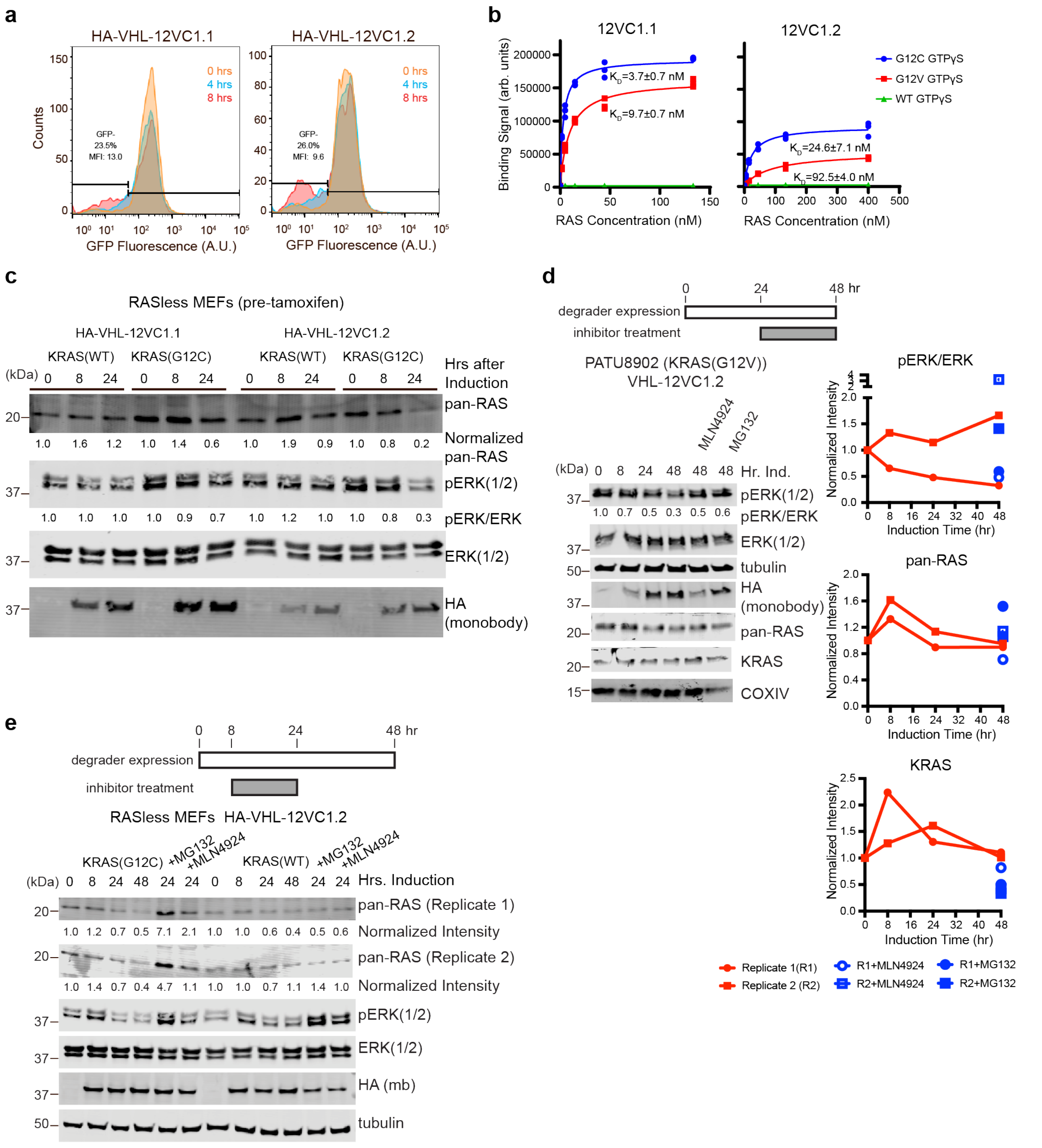

Supplementary Figure 10. Mutant-selective, noncovalent RAS degraders using monobody warheads. **a,** Degradation of EGFP-KRAS(G12C) in Flp293 cells expressing HA-VHL-12VC1.1 or 12VC1.2 under a dox-controlled expression system. Monobody clones, 12VC1.1 and 12VC1.2, were generated by replacing lysine residues of 12VC1 with glutamic acid. The level of KRAS was monitored at 0, 4, and 8 hours after dox induction using flow cytometry. 12VC1.2 degraded EGFP fused KRAS(G12C) more efficiently than 12VC1.1, as judged from the increase of the GFP-negative population. **b,** Binding titrations of 12VC1.1 and 1.2 with KRAS(G12C), KRAS(G12V), and KRAS(WT) bound to GTPγS using yeast display. The apparent K_D values of 12VC1.1 are 3.7 ± 0.7 nM and 9.7 ± 0.7 nM against G12C and G12V, respectively, and those of 12VC1.2 are 25 ± 7 nM and 93 ± 4 nM, respectively (mean and SD from n = 3, technical

replicates). Solid lines show the fits of the binding curve. **c,** The specificity and efficiency of VHL-monobody fusions tested in RASless MEFs ($KRAS^{flox/flox}$, $HRAS^{-/-}$, $NRAS^{-/-}$, with human KRAS(G12C) or KRAS(WT)). The cells were retrovirally transduced with a dox-inducible expression vector for HA-VHL-12VC1.1 or 12VC1.2. The abundance of KRAS and the pERK level were monitored at 0, 8, and 24 hours after induction with 1 µg/mL dox. Based on the initial results of **a** and **c**, it was decided that subsequent experiments targeting G12C would be performed using 12VC1.2 which generated reproducible results in terms of RAS degradation. **d,** Inefficient degradation of endogenous KRAS(G12V) in the PATU8902 cell line with VHL-12VC1.2. Total RAS, KRAS, and ERK activation were evaluated as a function of time. 12VC1.2, which was effective at degrading KRAS(G12C) was not efficient at degrading KRAS(G12V) (Biological replicates, *n* = 2). **e**, Degradation of KRAS(G12C) in RASless MEFs and effects of inhibitors of neddylation and proteasome degradation. A scheme of the experimental design is shown. The pan-RAS level was quantified with tubulin (technical replicate 1) or total ERK (technical replicate *n* = 2).

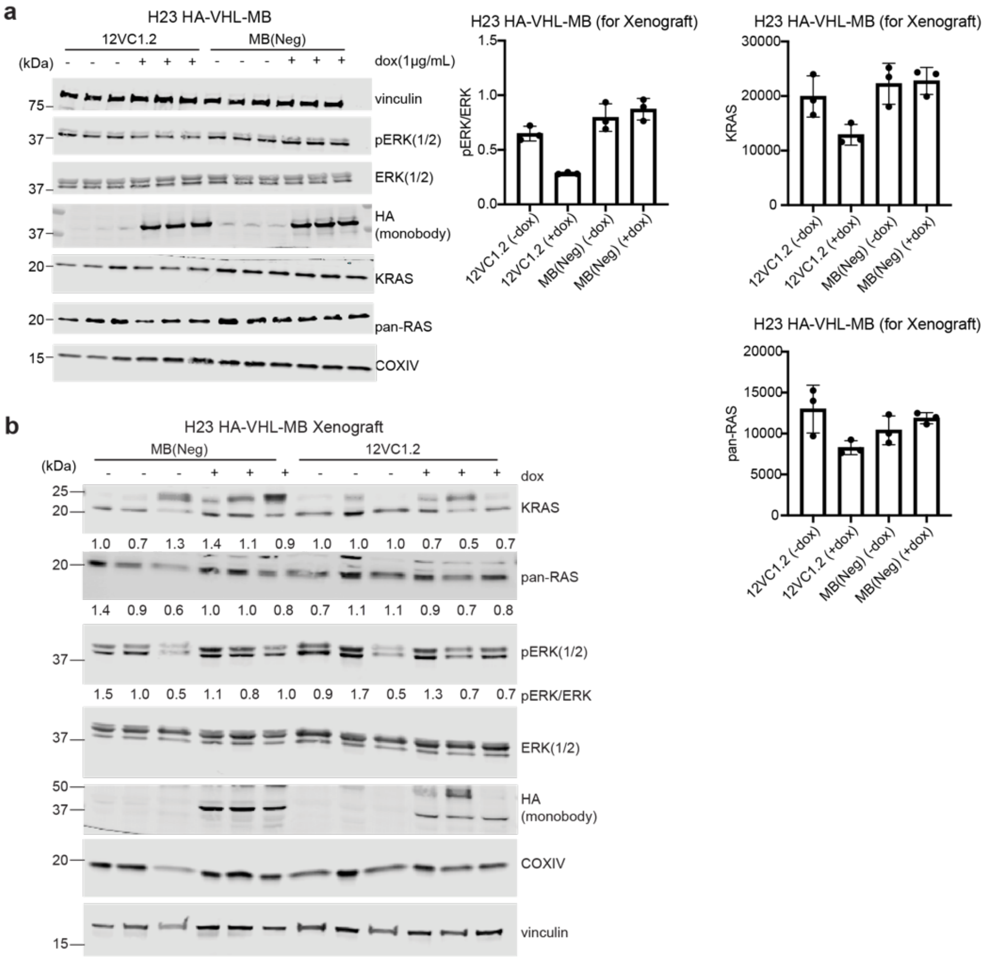

Supplementary Figure 11. Examination of the levels of relevant proteins in cell lines used for xenograft and those of extracted tumors by immunoblotting. **a**, The pERK, KRAS, and pan-RAS levels of the H23 cells that had been passaged by the same passage numbers as those used for xenograft experiments, with and without dox-induced monobody expression for 48 hours (biological replicates, *n* = 3). The goal was to ensure that the passaged cells did not lose the expression of monobodies due to high passage numbers necessary for producing large cell numbers. The pERK level was quantified using vinculin as a loading control, and the ratio of pERK to total ERK is reported. The pan-RAS and KRAS expression levels were quantified using COXIV as a loading control. The experiment was performed as biological triplicates. The mean and standard deviation of pERK/ERK, KRAS, and pan-RAS level are shown in the bar graphs (Mean $\pm$ SD). **b**, Immunoblotting of extracted tumors at the end of the xenograft experiment. Three tumors were randomly chosen for analysis from each treatment group with and without dox feeds. pERK was quantified using vinculin as a loading control. The ratio of pERK to ERK was normalized to the average of the pERK/ERK ratio from the 3 uninduced samples. Pan-RAS and KRAS levels were quantified using total ERK and COXVI respectively, and then normalized to the average RAS levels from the 3 uninduced samples. Level of VHL-monobody fusion (HA tag) was monitored using HA tag antibody. VHL-MB amounts were lower in the 12VC1.2-expressing samples at the end of the xenograft experiment when compared with MB(Neg)-expressing samples and the corresponding cell lines before implanting (shown in **a**).

a

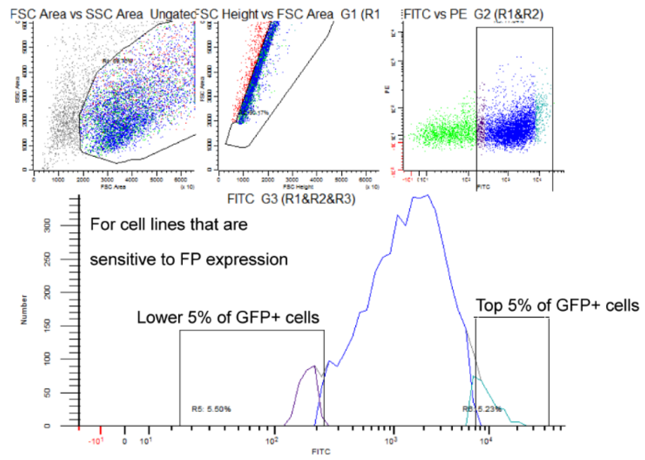

b

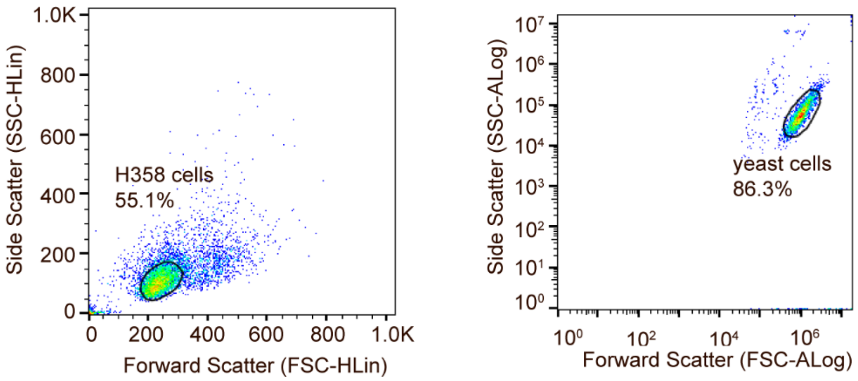

Supplementary Figure 12. Example gating strategies for flow cytometry experiments. **a**, Example gating strategy for sorting stable cell lines that express monobody fused to fluorescent protein (FP). Singlet population were gated based on SSC Height vs FSC Area (top middle panel). Fluorescence positive population (GFP or mVenus, FITC+) were gated based on comparison to non-fluorescence cells (top right panel). For cells that are insensitive to the expression of FP (HEK293T, H358, etc.), cells in this gate are collected and expanded for cell-based experiments. For cells that are sensitive to high expression level of FP, cells are sorted based on lower 5% of the FP+ population (bottom panel). **b**, Example gating strategy for analyzing mammalian and yeast cells. These experiments were performed using a single type of cells, which gave rise to a tight distribution on SSC vs FSC plot. This gate was applied before subsequent analysis of fluorescent population.

Supplementary References

1 Krissinel, E. & Henrick, K. Inference of macromolecular assemblies from crystalline state. *J Mol Biol* **372**, 774-797, doi:10.1016/j.jmb.2007.05.022 (2007).

2 Matsumoto, S. *et al.* Molecular Mechanism for Conformational Dynamics of Ras.GTP Elucidated from In-Situ Structural Transition in Crystal. *Sci Rep* **6**, 25931, doi:10.1038/srep25931 (2016).

3 Muraoka, S. *et al.* Crystal structures of the state 1 conformations of the GTP-bound H-Ras protein and its oncogenic G12V and Q61L mutants. *FEBS Lett* **586**, 1715-1718, doi:10.1016/j.febslet.2012.04.058 (2012).

4 Fetics, S. K. *et al.* Allosteric effects of the oncogenic RasQ61L mutant on Raf-RBD. *Structure* **23**, 505-516, doi:10.1016/j.str.2014.12.017 (2015).

5 Ostrem, J. M., Peters, U., Sos, M. L., Wells, J. A. & Shokat, K. M. K-Ras(G12C) inhibitors allosterically control GTP affinity and effector interactions. *Nature* **503**, 548-551, doi:10.1038/nature12796 (2013).

6 Hall, B. E., Bar-Sagi, D. & Nassar, N. The structural basis for the transition from Ras-GTP to Ras-GDP. *Proc Natl Acad Sci U S A* **99**, 12138-12142, doi:10.1073/pnas.192453199 (2002).

7 Huang, L., Hofer, F., Martin, G. S. & Kim, S. H. Structural basis for the interaction of Ras with RalGDS. *Nat Struct Biol* **5**, 422-426, doi:10.1038/nsb0698-422 (1998).

8 Buhrman, G., de Serrano, V. & Mattos, C. Organic solvents order the dynamic switch II in Ras crystals. *Structure* **11**, 747-751, doi:10.1016/s0969-2126(03)00128-x (2003).

9 Ford, B., Hornak, V., Kleinman, H. & Nassar, N. Structure of a transient intermediate for GTP hydrolysis by ras. *Structure* **14**, 427-436, doi:10.1016/j.str.2005.12.010 (2006).

10 Bunney, T. D. *et al.* Structural and mechanistic insights into ras association domains of phospholipase C epsilon. *Mol Cell* **21**, 495-507, doi:10.1016/j.molcel.2006.01.008 (2006).

11 Buhrman, G., Wink, G. & Mattos, C. Transformation efficiency of RasQ61 mutants linked to structural features of the switch regions in the presence of Raf. *Structure* **15**, 1618-1629, doi:10.1016/j.str.2007.10.011 (2007).

12 Tanaka, T. & Rabbitts, T. H. Functional intracellular antibody fragments do not require invariant intra-domain disulfide bonds. *J Mol Biol* **376**, 749-757, doi:S0022-2836(07)01583-5 [pii]10.1016/j.jmb.2007.11.085 (2008).

13 Stieglitz, B. *et al.* Novel type of Ras effector interaction established between tumour suppressor NORE1A and Ras switch II. *EMBO J* **27**, 1995-2005, doi:10.1038/emboj.2008.125 (2008).

14 Shima, F. *et al.* Structural basis for conformational dynamics of GTP-bound Ras protein. *J Biol Chem* **285**, 22696-22705, doi:10.1074/jbc.M110.125161 (2010).

15 Rosnizeck, I. C. *et al.* Stabilizing a weak binding state for effectors in the human ras protein by cyclen complexes. *Angew Chem Int Ed Engl* **49**, 3830-3833, doi:10.1002/anie.200907002 (2010).

16 Buhrman, G., Kumar, V. S., Cirit, M., Haugh, J. M. & Mattos, C. Allosteric modulation of Ras-GTP is linked to signal transduction through RAF kinase. *J Biol Chem* **286**, 3323-3331, doi:10.1074/jbc.M110.193854 (2011).

17 Buhrman, G. *et al.* Analysis of binding site hot spots on the surface of Ras GTPase. *J Mol Biol* **413**, 773-789, doi:10.1016/j.jmb.2011.09.011 (2011).

18 Fraser, J. S. *et al.* Accessing protein conformational ensembles using room-temperature X-ray crystallography. *Proc Natl Acad Sci U S A* **108**, 16247-16252, doi:10.1073/pnas.1111325108 (2011).

19 Holzapfel, G., Buhrman, G. & Mattos, C. Shift in the equilibrium between on and off states of the allosteric switch in Ras-GppNHp affected by small molecules and bulk solvent composition. *Biochemistry* **51**, 6114-6126, doi:10.1021/bi300509j (2012).

20 Qamra, R. & Hubbard, S. R. Structural basis for the interaction of the adaptor protein grb14 with activated ras. *PLoS One* **8**, e72473, doi:10.1371/journal.pone.0072473 (2013).
21 Bandaru, P. *et al.* Deconstruction of the Ras switching cycle through saturation mutagenesis. *Elife* **6**, doi:10.7554/eLife.27810 (2017).
22 Ke, H. *et al.* Structural basis for intramolecular interaction of post-translationally modified H-Ras*GTP prepared by protein ligation. *FEBS Lett* **591**, 2470-2481, doi:10.1002/1873-3468.12759 (2017).
23 Smith, M. J. *et al.* Evolution of AF6-RAS association and its implications in mixed-lineage leukemia. *Nat Commun* **8**, 1099, doi:10.1038/s41467-017-01326-5 (2017).
24 Abbott, J. R. *et al.* Discovery of Quinazolines That Activate SOS1-Mediated Nucleotide Exchange on RAS. *ACS Med Chem Lett* **9**, 941-946, doi:10.1021/acsmedchemlett.8b00296 (2018).
25 Milburn, M. V. *et al.* Molecular switch for signal transduction: structural differences between active and inactive forms of protooncogenic ras proteins. *Science* **247**, 939-945, doi:10.1126/science.2406906 (1990).
26 Rooklin, D., Wang, C., Katigbak, J., Arora, P. S. & Zhang, Y. AlphaSpace: Fragment-Centric Topographical Mapping To Target Protein-Protein Interaction Interfaces. *J Chem Inf Model* **55**, 1585-1599, doi:10.1021/acs.jcim.5b00103 (2015).
27 McDonald, E. R., 3rd *et al.* Project DRIVE: A Compendium of Cancer Dependencies and Synthetic Lethal Relationships Uncovered by Large-Scale, Deep RNAi Screening. *Cell* **170**, 577-592 e510, doi:10.1016/j.cell.2017.07.005 (2017).
