# Supplementary material for: Selective and noncovalent targeting of RAS mutants for inhibition and degradation
Source: Nat Commun. 2021 May 11;12:2656. doi: 10.1038/s41467-021-22969-5 (PMC8113534; doi:10.1038/s41467-021-22969-5)
Supplement: Supplementary file 3 — Reporting Summary [file 41467_2021_22969_MOESM3_ESM.pdf]

nature research

Corresponding author(s): Shohei Koide
Last updated by author(s): Mar 9, 2021

Reporting Summary

Nature Research wishes to improve the reproducibility of the work that we publish. This form provides structure for consistency and transparency in reporting. For further information on Nature Research policies, see our Editorial Policies and the Editorial Policy Checklist.

Statistics

For all statistical analyses, confirm that the following items are present in the figure legend, table legend, main text, or Methods section.

n/a	Confirmed	
☐	☒	The exact sample size (*n*) for each experimental group/condition, given as a discrete number and unit of measurement
☐	☒	A statement on whether measurements were taken from distinct samples or whether the same sample was measured repeatedly
☐	☒	The statistical test(s) used AND whether they are one- or two-sided<br>*Only common tests should be described solely by name; describe more complex techniques in the Methods section.*
☐	☒	A description of all covariates tested
☐	☒	A description of any assumptions or corrections, such as tests of normality and adjustment for multiple comparisons
☐	☒	A full description of the statistical parameters including central tendency (e.g. means) or other basic estimates (e.g. regression coefficient) AND variation (e.g. standard deviation) or associated estimates of uncertainty (e.g. confidence intervals)
☐	☒	For null hypothesis testing, the test statistic (e.g. *F*, *t*, *r*) with confidence intervals, effect sizes, degrees of freedom and *P* value noted<br>*Give P values as exact values whenever suitable.*
☒	☐	For Bayesian analysis, information on the choice of priors and Markov chain Monte Carlo settings
☒	☐	For hierarchical and complex designs, identification of the appropriate level for tests and full reporting of outcomes
☒	☐	Estimates of effect sizes (e.g. Cohen's *d*, Pearson's *r*), indicating how they were calculated

Our web collection on statistics for biologists contains articles on many of the points above.

Software and code

Policy information about availability of computer code

Data collection	ForeCyte v8.0, GuavaSoft v2.1, Octet Data Acquisition, Zeiss ZEN Software 2010. Thermal Fisher Q Exactive Mass Spectrometer Software. All softwares are from third party developers.
Data analysis	Octet Data Analysis version 9.0.0.14, Prism v8, FlowJo V9.9.6, HKL3000, PHENIX v1.13-2998-000, Phaser version 2.8.1, Coot version 0.8.9, ImageJ v1.52P, Image Studio Lite v5.2, PyMol v2.3.2. Proteome Discoverer 1.4.0.288. Amber Package 18.04. All softwares are from third party developers.

For manuscripts utilizing custom algorithms or software that are central to the research but not yet described in published literature, software must be made available to editors and reviewers. We strongly encourage code deposition in a community repository (e.g. GitHub). See the Nature Research guidelines for submitting code & software for further information.

Data

Policy information about availability of data

All manuscripts must include a data availability statement. This statement should provide the following information, where applicable:

- Accession codes, unique identifiers, or web links for publicly available datasets
- A list of figures that have associated raw data
- A description of any restrictions on data availability

Crystallography and sequence data for unique reagents that were generated in this study have been deposited in the Protein Data Bank (https://www.rcsb.org/) under PDB accession codes 7L0G and 7L0F. Uniprot homo sapiens reference database (https://www.uniprot.org/proteomes/UP000005640) has been used in the study for identifying mass spectrometry species. Mass spectrometry raw data have been deposited onto the Mass Spectrometry Interactive Virtual Environment (https://massive.ucsd.edu/ProteoSAFe/static/massive.jsp) under MassIVE ID MSV000086569. The uncropped version of Western blots from this study has been provided. Raw data generated from this study are available upon a reasonable request.

Field-specific reporting

Please select the one below that is the best fit for your research. If you are not sure, read the appropriate sections before making your selection.

☒ Life sciences ☐ Behavioural & social sciences ☐ Ecological, evolutionary & environmental sciences

For a reference copy of the document with all sections, see nature.com/documents/nr-reporting-summary-flat.pdf

Life sciences study design

All studies must disclose on these points even when the disclosure is negative.

Sample size	The sample size for curve fitting of the yeast display binding data was determined based on our extensive experience that the data points follow the 1:1 binding model and replicates have small variations. The sample size for signaling assays was determined based on observation of consistent data through multiple biological replicates. The sample size for cell-based assays was determined based on the number of experimental samples that can be reasonably handled in a multi-well plate and in a 15-well gel, and whether replicates follow consistent results. The sample size for mouse xenograft studies was determined based on the capacity of mice that one cage can hold (5) and "resource equation" (PMID: 24250214), where the quantity E = total number of animals - number of treatment groups should be between 10 to 20. For example, 4-5 animals per group in a 4-arm experiment yields E=12 to 16.
Data exclusions	No data were excluded in the study except in one instance where we excluded a mouse that had died prior to the beginning of the experimental treatment.
Replication	All experiment were performed as duplicate at minimum in succession unless otherwise stated.
Randomization	Samples were randomly drawn from the main population for experiment.
Blinding	Blinding was applied during tumor dissection to avoid bias. Blinding was not applied for any other experiments where data acquisition were automatically performed by instrument to avoid bias. For other experiments where samples were processed manually, e.g., western blots and cell-based flow cytometry assays, blinding was not performed due to 1) avoidance of mishandling samples, 2) more than one condition that demonstrated a trend in response to a condition (e.g., titration of doxycycline or time course experiment) and 3) the magnitude of experimental responses was significant to warrant that changes observed were not possible to be an outcome of a slight bias.

Reporting for specific materials, systems and methods

We require information from authors about some types of materials, experimental systems and methods used in many studies. Here, indicate whether each material, system or method listed is relevant to your study. If you are not sure if a list item applies to your research, read the appropriate section before selecting a response.

Materials & experimental systems

n/a	Involved in the study	
☐	☒	Antibodies
☐	☒	Eukaryotic cell lines
☒	☐	Palaeontology and archaeology
☐	☒	Animals and other organisms
☒	☐	Human research participants
☒	☐	Clinical data
☒	☐	Dual use research of concern

Methods

n/a	Involved in the study	
☒	☐	ChIP-seq
☐	☒	Flow cytometry
☒	☐	MRI-based neuroimaging

Antibodies

Antibodies used	Mouse anti-pan-RAS(SCBT, cat#166691, clone C-4), Rabbit anti-phospho-p44/42 MAPK ERK1/2 (Cell Signaling Technology, cat#9101), Rabbit anti-p44/42 MAPK ERK1/2(Cell Signaling Technology, cat#9102), Mouse anti-alpha-tubulin (ThermoFisher Scientific, cat#62204, clone DM1A), Mouse anti-HA-tag (BioLegends, cat#901516, clone 16B12), Mouse anti-FLAG-tag (Sigma, cat#F3165, clone M2), Mouse anti-V5 tag antibody (ThermoFisher, cat#MA5-15253, clone E10/V4RR), Goat anti-Mouse antibody FITC conjugate (Millipore Sigma, cat#F0257), KRAS antibody (Sigma Aldrich, cat#WH0003845M1), COX IV antibody (Li-Cor, cat# 926-42214), Vinculin antibody (ThermalFisher, cat#700062). IRDye 680LT Goat anti-Mouse IgG secondary (926-68020), IRDye 800CW Goat anti-Rabbit IgG secondary antibody (926-32211). Goat Anti-mouse IgG Cross-Absorbed secondary antibody, HRP (pierce, cat#31432).
Validation	Mouse anti-pan RAS(SCBT, cat#166691, clone C-4) is a monoclonal antibody for the detection of human, mouse, rat pan-RAS protein. The antibody has been cited by 25 citations (e.g. PMID: 31182717). The antibody successfully detected the presence of KRAS in transfected HEK293 cells over-expressing human KRAS protein relative to the non-transfected counterpart. The manufacturer's website (https://www.scbt.com/p/pan-ras-antibody-c-4) provides additional antibody information and citations.

Rabbit anti-phospho-p44/42 MAPK ERK1/2 (Cell Signaling Technology, cat#9101) is a polyclonal antibody against phosphorylated form of ERK1/2 with cross-species reactivity in human, mouse, and rats etc. The antibody has over 4000 citations. Validation statements and citations are provided on the manufacturer's website (https://www.cellsignal.com/products/primary-antibodies/phospho-p44-42-mapk-erk1-2-thr202-tyr204-antibody/9101).

Rabbit anti-p44/42 MAPK ERK1/2 (Cell Signaling Technology, cat#9102) is a polyclonal antibody against ERK1/2 with over 4000 citations. There is cross-species reactivity to human, mouse, and rats ERK. Validation statements and citations are provided on the manufacturer's website (https://www.cellsignal.com/products/primary-antibodies/p44-42-mapk-erk1-2-antibody/9102).

Mouse anti-alpha-tubulin (ThermoFisher Scientific, cat#62204, clone DM1A, is a monoclonal antibody against alpha-tubulin. The product has over 100 citations. The product has cross-species reactivity towards human, mouse, and rats. Validation statement and citations are provided on the manufacturer's website.

Mouse anti-HA-tag (BioLegends, cat#901516, clone 16B12) is a monoclonal antibody against HA epitope tag (YPYDVPDYA) with over 140 citations. The antibody detected HA tagged protein in CHO cells stably expressing HA-tagged protein, and no band was observed from lysates of regular CHO cells. Validation statements and citations are provided on the manufacturer's website.

Mouse anti-Flag-tag (Sigma, cat#F3165, clone M2) is a monoclonal antibody against FLAG epitope tag (DYKDDDDK). The product has over 5000 citations. Validation statements and citations are provided on the manufacturer's website.

Mouse anti-V5 tag antibody (ThermoFisher, cat#MA5-15253, clone E10/V4RR) is a monoclonal antibody against V5 tag. The product has over 30 citations. The antibody detected V5 protein in cells transfected with plasmids encoding for V5 tagged protein, and no band was observed from the control cell lysates. Validation statements and citations are provided on the manufacturer's website.

Goat anti-Mouse antibody FITC conjugate (Millipore Sigma, cat#F0257) is a polyclonal antibody against mouse IgG. The product has 150 citations. Validation statements and citations are provided on the manufacturer's website.

KRAS antibody (Sigma Aldrich, cat#WH0003845M1) is a mouse monoclonal antibody against KRAS. The product has over 40 citations (notably, see PMID:27338794, PMID: 31827279). The antibody has been validated in this study PMID: 28951536. Citations are provided on the manufacturer's website.

COX IV antibody (Li-Cor, cat#926-42214) is rabbit monoclonal antibody against cytochrome oxidase c, used as a loading control. It has cross-species reactivity against human, rabbit, monkey, zebra fish.

Vinculin antibody (ThermoFisher Scientific, cat#700062) is a recombinant rabbit monoclonal antibody against vinculin, used as a loading control. The antibody has been validated by relative expression technique to ensure that the antibody binds to the stated antigen. The product currently has over 20 citations. Additional validation statement and citations are provided on the manufacturer's website.

Information on secondary antibodies can be found on their respective websites.

Eukaryotic cell lines

Policy information about cell lines

Cell line source(s)	HPAF-II, H441, A375, and HEK293T were purchased directly from ATCC. RASlessMEFs were from the National Cancer Institute RAS Initiative at the Frederick National Laboratory. PATU8902 was from Dr. Alec Kimmelman (NYU School of Medicine; July 2017). H358 and H23 were from Dr. Kwok-Kin Wong (NYU School of Medicine; July 2017). Cells obtained from collaborators were genotyped by STR analysis at IDEXX Bioresearch (December 2017).
Authentication	PATU8902, H358, H23 were authenticated by IDEXX using STR analysis. Cell lines directly purchased from ATCC (HPAF-II, H441, A375, HEK293T) were authenticated by ATCC using morphology, karyotyping or PCR methods.
Mycoplasma contamination	All cell lines were tested for mycoplasma contamination using the e-Myco Plus PCR detection kit, and cultured in the presence of antimycotic/antibiotic unless otherwise stated. The results of all PCR tests performed on the cell lines stated above were negative.
Commonly misidentified lines (See ICLAC register)	No commonly misidentified cell lines were used.

Animals and other organisms

Policy information about studies involving animals; ARRIVE guidelines recommended for reporting animal research

Laboratory animals	Mouse, strain: CR ATH HO (490), females approx. 8 weeks of age.
Wild animals	The study did not involve wild animals.
Field-collected samples	The study did not involve field collected samples.

Ethics oversight	The study was approved by IACUC of NYU Langone under the protocol number of IACUC, protocol 170602.

Note that full information on the approval of the study protocol must also be provided in the manuscript.

Flow Cytometry

Plots

Confirm that:

- [x] The axis labels state the marker and fluorochrome used (e.g. CD4-FITC).
- [x] The axis scales are clearly visible. Include numbers along axes only for bottom left plot of group (a 'group' is an analysis of identical markers).
- [x] All plots are contour plots with outliers or pseudocolor plots.
- [x] A numerical value for number of cells or percentage (with statistics) is provided.

Methodology

Sample preparation	100,000 yeast cells per clone were incubated with 100x dilution of anti-V5 tag antibody and biotinylated target at 2x the given concentration in a final 20 uL volume for 30 minutes at room temperature in BSS buffer (50 mM Tris-Cl, 150 mM NaCl, pH8) supplemented with 5 mg/mL BSA, 20 mM MgCl2, 0.1 mM DTT in a 96-well U bottom plate (Greiner 650201) while shaking. After incubation, the yeast cells were transfered to a 96-well filter plate (EMD Millipore MSHVN4550). The yeasts cells were washed twice with approximately 150 uL of supplemented BSS buffer with 0.1% tween-20 (BSST). Buffer were withdrawn from the wells by a vacuum. Yeast cells were then stained with anti-mouse FITC and Neutravidin Dylight 650 (10ug/mL) in 20 uL final volume at 4 degree C while shaking. Wells were washed 2x with supplemented BSST. Final suspension were made with 100 uL of suplemented BSS right before measurement.<br>Mammalian cells used in this study were all adherent and grown on multi-well cell culture plates before measurement. Cells were washed 1x with PBS before incubating with trypsin (trypLE) for detachment. Cells were incubated in 5% CO2 incubator for 5-10 minutes until they completely detach. Cells were then neutralized with media at ~5x excess volume. Cells were spun down in a centrifuge for 2 minutes at 500g. Supernatant were then removed. Cell pellet were suspended in 1mg/mL BSA, DPBS for flow cytometry measurement. No cell staining was applied, as we only detected intracellular fluorescent proteins.
Instrument	Intellicyt Hypercyt (Sartorius), Intellicyt iQue3 (Sartorius), Guava easyCyte BGR (EMD Millipore)
Software	Data were collected using ForeCyte on the Intellicyt instruments, and GuavaSoft on the Guava instrument.
Cell population abundance	Expression of monobody on yeast cells was approximately 50% based on labeling of V5 tag with FITC at the C terminus of mono body.<br>For dox-inducible experiments, the percentage of population expressing monobody was determined based on the fluorescence emission of fluorescent proteins, EGFP or mVenus. Cells expressing monobody were 75%-100% of the population based on this assessment.
Gating strategy	Yeast cells were gated on the contour plot of FSC vs SSC based on the major distribution of size events. Yeast cells have a tight distribution on the FSC vs SSC plot. Binding signal was measured by taking the median fluorescence intensity (MFI) of the 75-95th percentile fluorescent population in the Dylight 650 channel.<br>For mammalian cells, cells were first gated on FSC/SSC based on major distribution of size events that is above the thresh hold of the background debris/particles. For each cell line, the FCS/SSC profile varies, and the gate was adjusted accordingly. The fluorescence-positive population was gated using the distribution of fluorescence intensity of the uninduced cells as a cut off (SI Fig. 7c).

- [x] Tick this box to confirm that a figure exemplifying the gating strategy is provided in the Supplementary Information.
