# Supplementary material for: Selective and noncovalent targeting of RAS mutants for inhibition and degradation
Source: Nat Commun. 2021 May 11;12:2656. doi: 10.1038/s41467-021-22969-5 (PMC8113534; doi:10.1038/s41467-021-22969-5)
Supplement: Supplementary file 4 — Source Data [file 41467_2021_22969_MOESM4_ESM.zip › 282021_2_related_ms_5410672_qq17ll.pptx]

## Slide 1
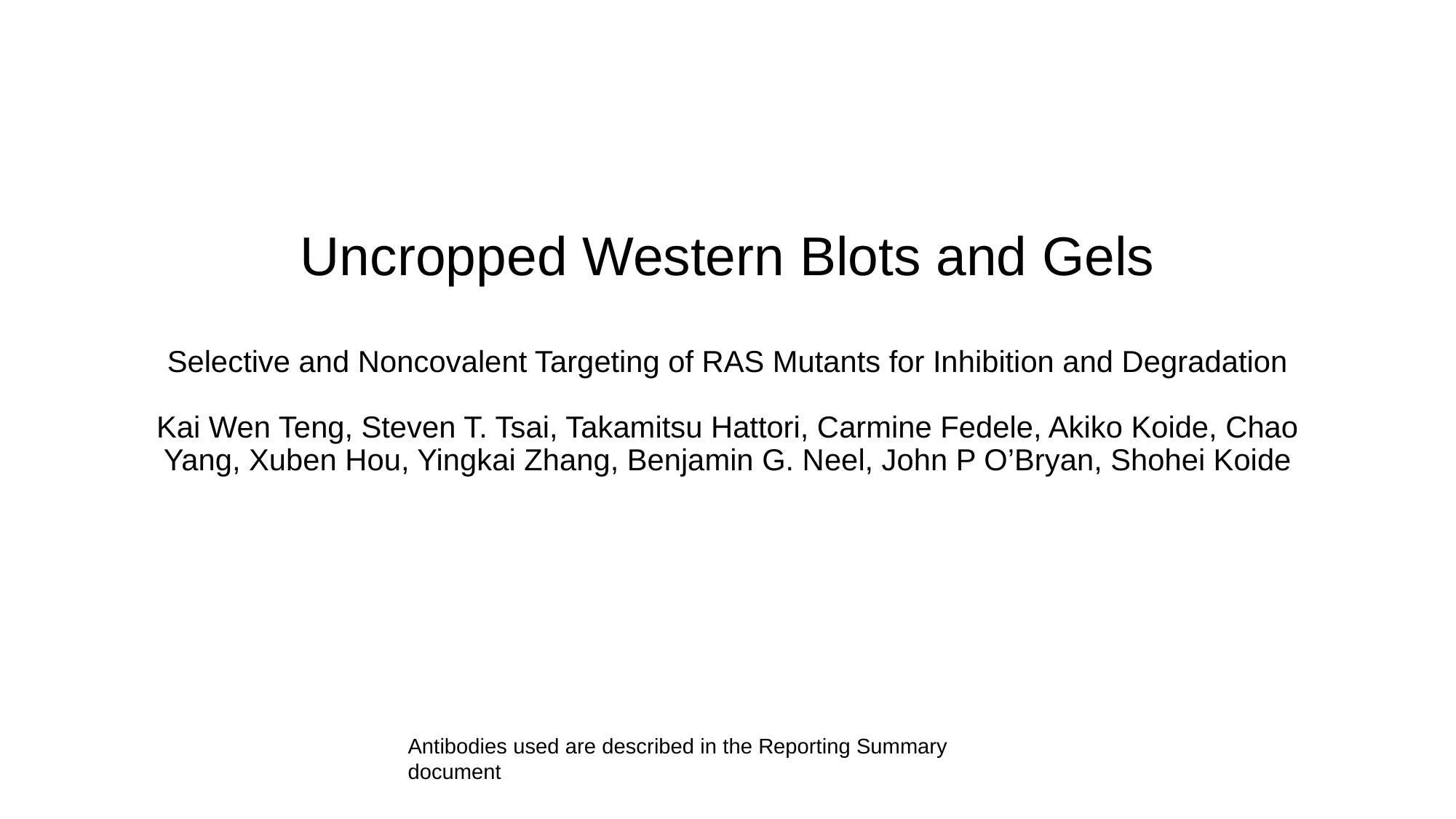

# Uncropped Western Blots and Gels Selective and Noncovalent Targeting of RAS Mutants for Inhibition and Degradation Kai Wen Teng, Steven T. Tsai, Takamitsu Hattori, Carmine Fedele, Akiko Koide, Chao Yang, Xuben Hou, Yingkai Zhang, Benjamin G. Neel, John P O’Bryan, Shohei Koide
Antibodies used are described in the Reporting Summary document

## Slide 2
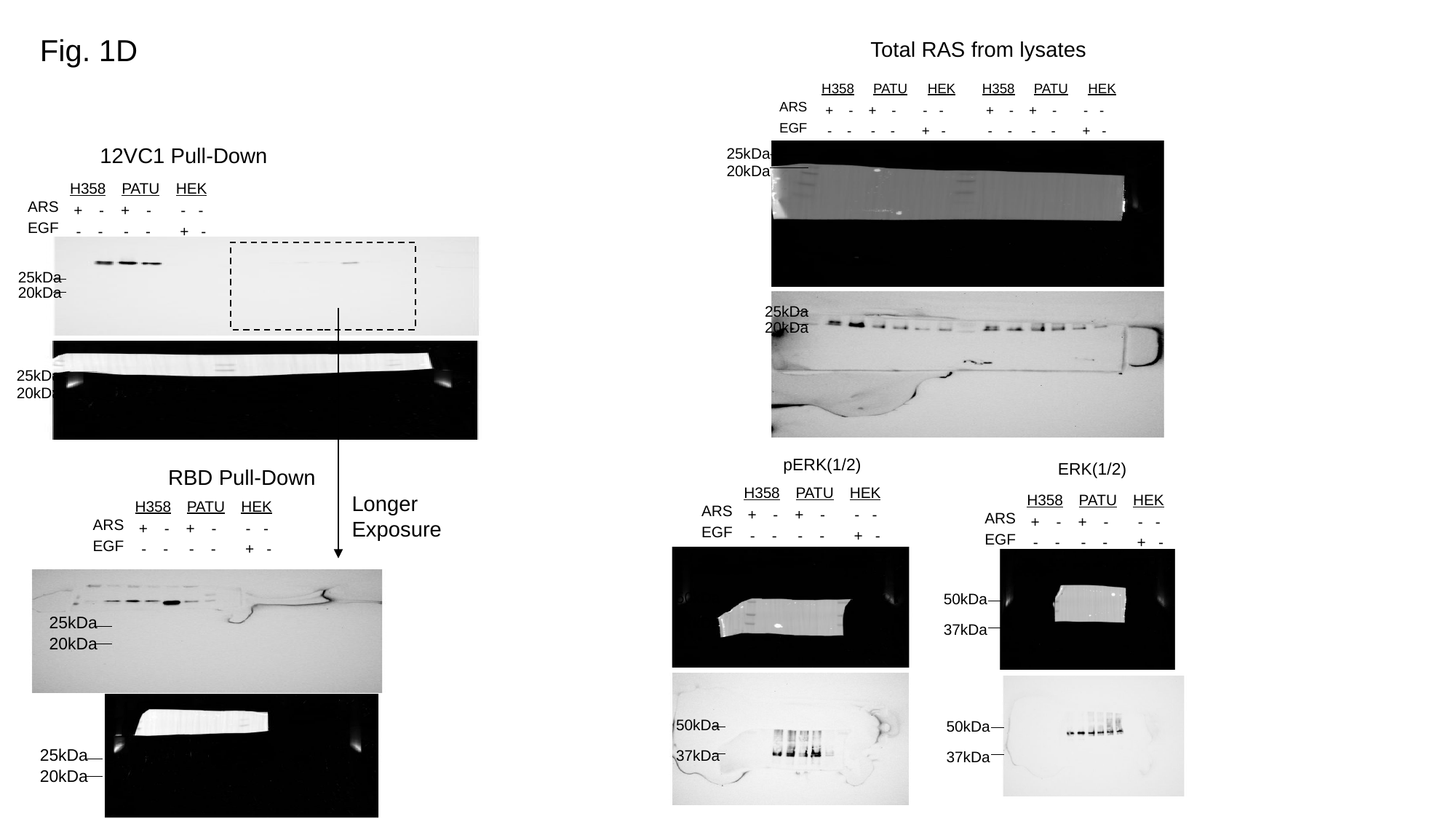

# Fig. 1D
Total RAS from lysates
H358
PATU
HEK
H358
PATU
HEK
ARS
+ - + - - -
+ - + - - -
EGF
 - - - - + -
 - - - - + -
12VC1 Pull-Down
25kDa
20kDa
25kDa
20kDa
H358
PATU
HEK
ARS
+ - + - - -
EGF
 - - - - + -
25kDa
20kDa
25kDa
20kDa
pERK(1/2)
ERK(1/2)
RBD Pull-Down
H358
PATU
HEK
Longer Exposure
H358
PATU
HEK
H358
PATU
HEK
ARS
+ - + - - -
ARS
+ - + - - -
ARS
+ - + - - -
EGF
 - - - - + -
EGF
 - - - - + -
EGF
 - - - - + -
50kDa
37kDa
25kDa
20kDa
50kDa
37kDa
25kDa
20kDa
50kDa
50kDa
37kDa
37kDa

## Slide 3
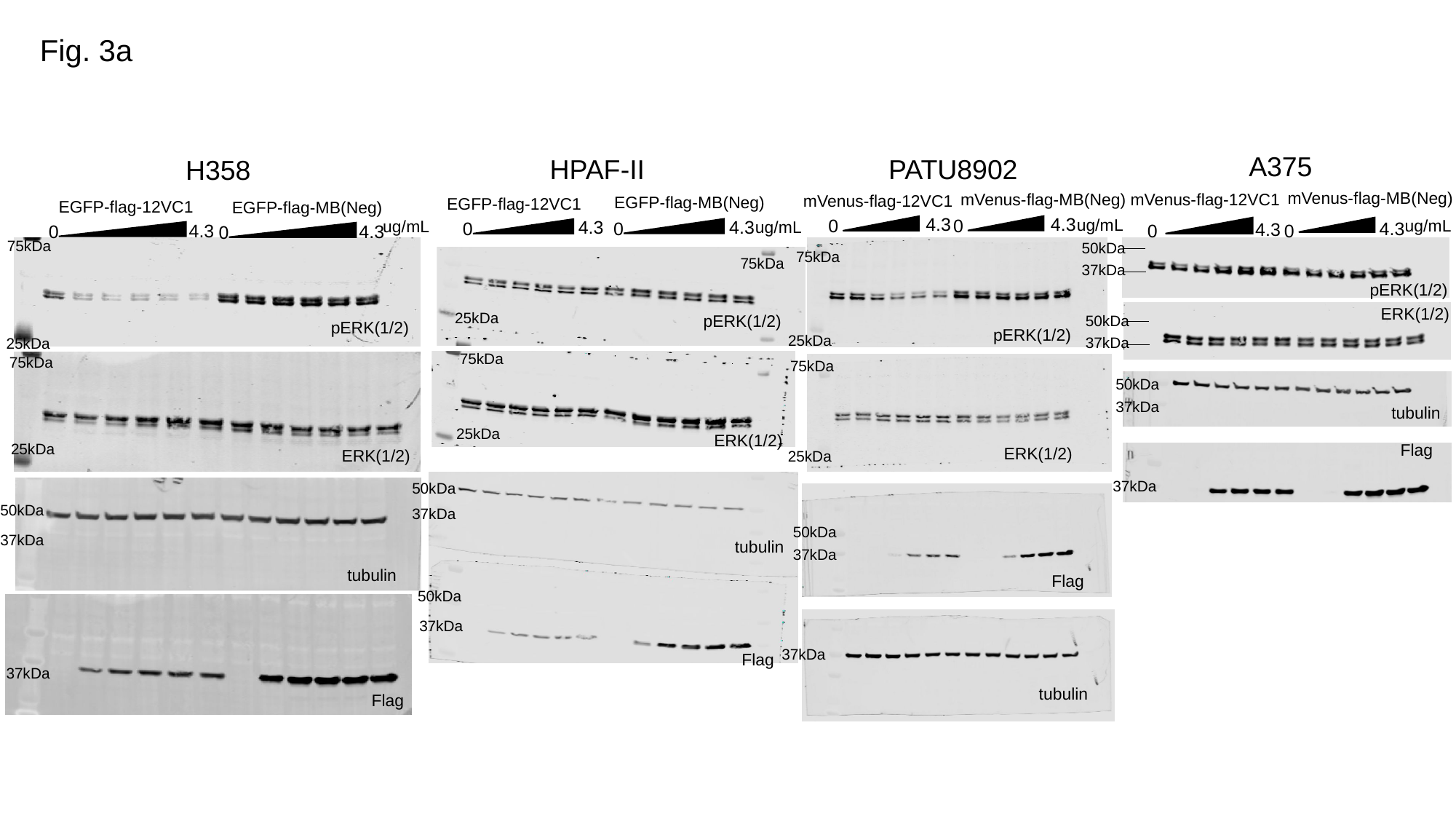

# Fig. 3a
A375
HPAF-II
PATU8902
H358
mVenus-flag-MB(Neg)
mVenus-flag-12VC1
mVenus-flag-MB(Neg)
mVenus-flag-12VC1
EGFP-flag-MB(Neg)
EGFP-flag-12VC1
EGFP-flag-12VC1
EGFP-flag-MB(Neg)
4.3
4.3
0
ug/mL
0
ug/mL
4.3
4.3
ug/mL
ug/mL
4.3
0
0
4.3
0
0
4.3
0
4.3
0
75kDa
50kDa
75kDa
75kDa
37kDa
pERK(1/2)
ERK(1/2)
25kDa
pERK(1/2)
50kDa
pERK(1/2)
pERK(1/2)
25kDa
37kDa
25kDa
75kDa
75kDa
75kDa
50kDa
37kDa
tubulin
25kDa
ERK(1/2)
25kDa
Flag
ERK(1/2)
ERK(1/2)
25kDa
37kDa
50kDa
50kDa
37kDa
50kDa
37kDa
tubulin
37kDa
tubulin
Flag
50kDa
37kDa
37kDa
Flag
37kDa
tubulin
Flag

## Slide 4
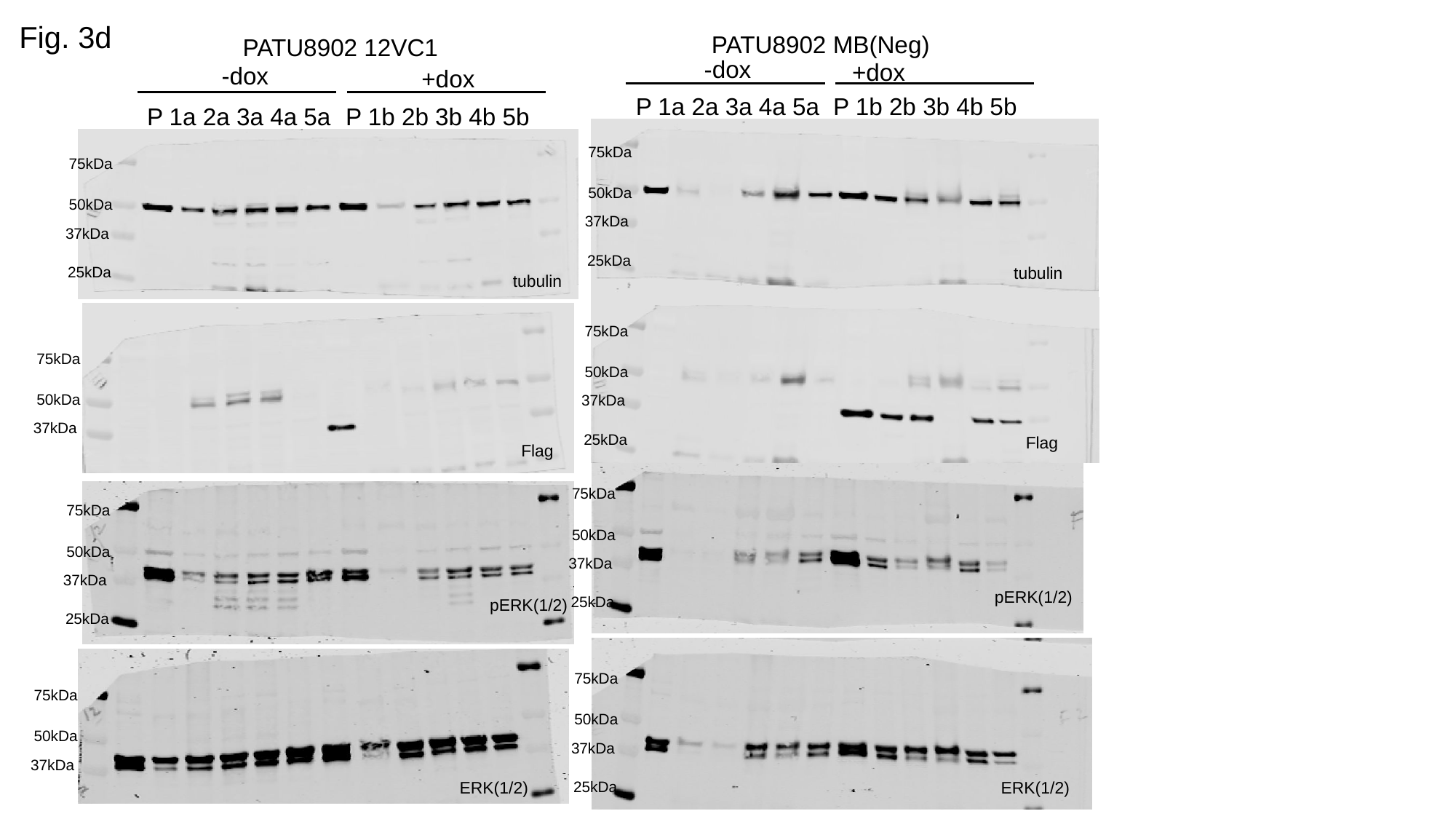

Fig. 3d
PATU8902 MB(Neg)
PATU8902 12VC1
-dox
+dox
-dox
+dox
P 1b 2b 3b 4b 5b
P 1a 2a 3a 4a 5a
P 1b 2b 3b 4b 5b
P 1a 2a 3a 4a 5a
75kDa
75kDa
50kDa
50kDa
37kDa
37kDa
25kDa
25kDa
tubulin
tubulin
75kDa
75kDa
50kDa
50kDa
37kDa
37kDa
25kDa
Flag
Flag
75kDa
75kDa
50kDa
50kDa
37kDa
37kDa
pERK(1/2)
25kDa
pERK(1/2)
25kDa
75kDa
75kDa
50kDa
50kDa
37kDa
37kDa
25kDa
ERK(1/2)
ERK(1/2)

## Slide 5
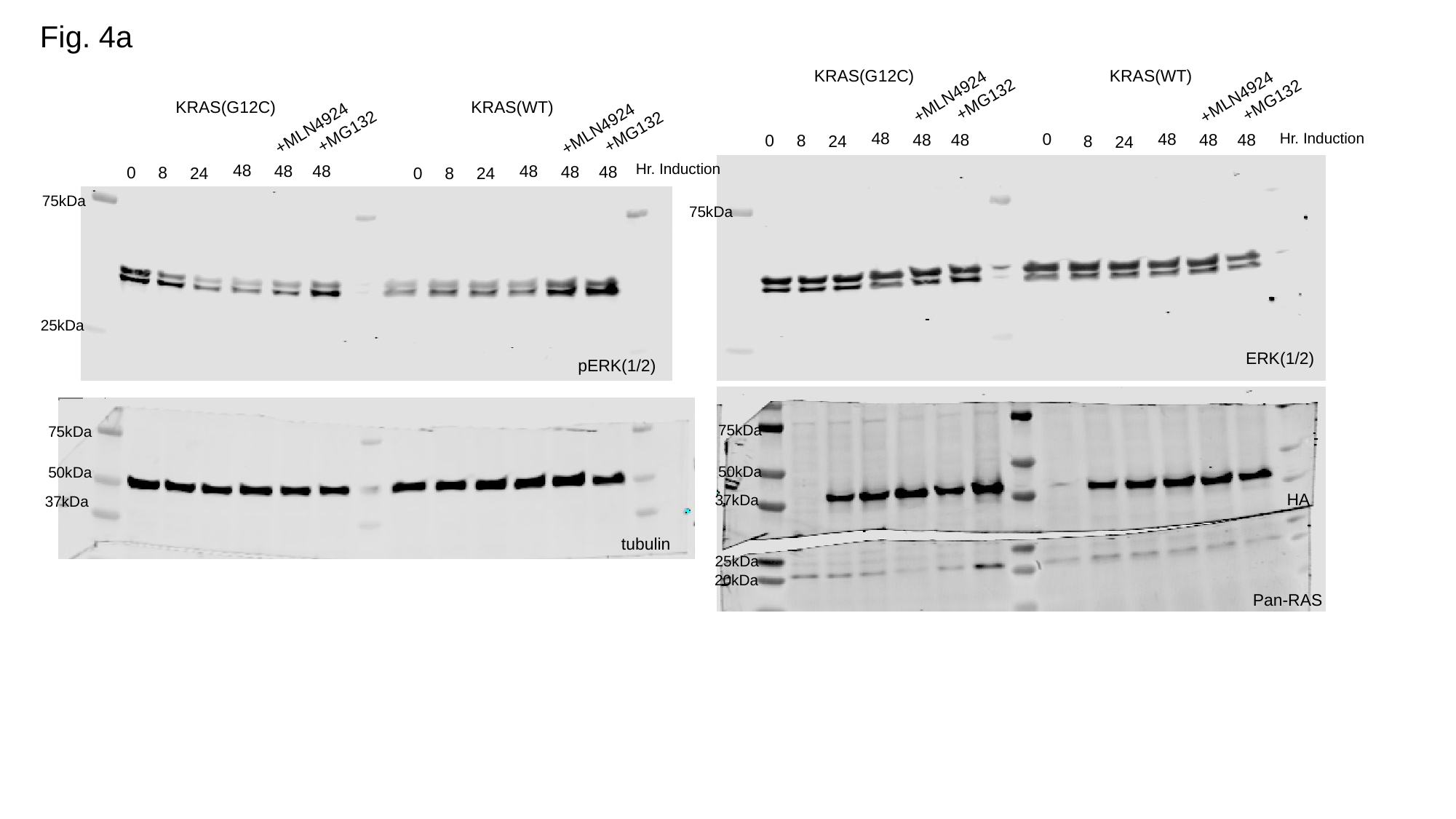

Fig. 4a
KRAS(G12C)
KRAS(WT)
+MLN4924
+MLN4924
+MG132
+MG132
KRAS(G12C)
KRAS(WT)
+MLN4924
+MLN4924
+MG132
+MG132
48
Hr. Induction
0
48
48
48
48
48
0
8
24
8
24
Hr. Induction
48
48
48
48
48
48
0
8
24
0
8
24
75kDa
75kDa
25kDa
ERK(1/2)
pERK(1/2)
75kDa
75kDa
50kDa
50kDa
HA
37kDa
37kDa
tubulin
25kDa
20kDa
Pan-RAS

## Slide 6
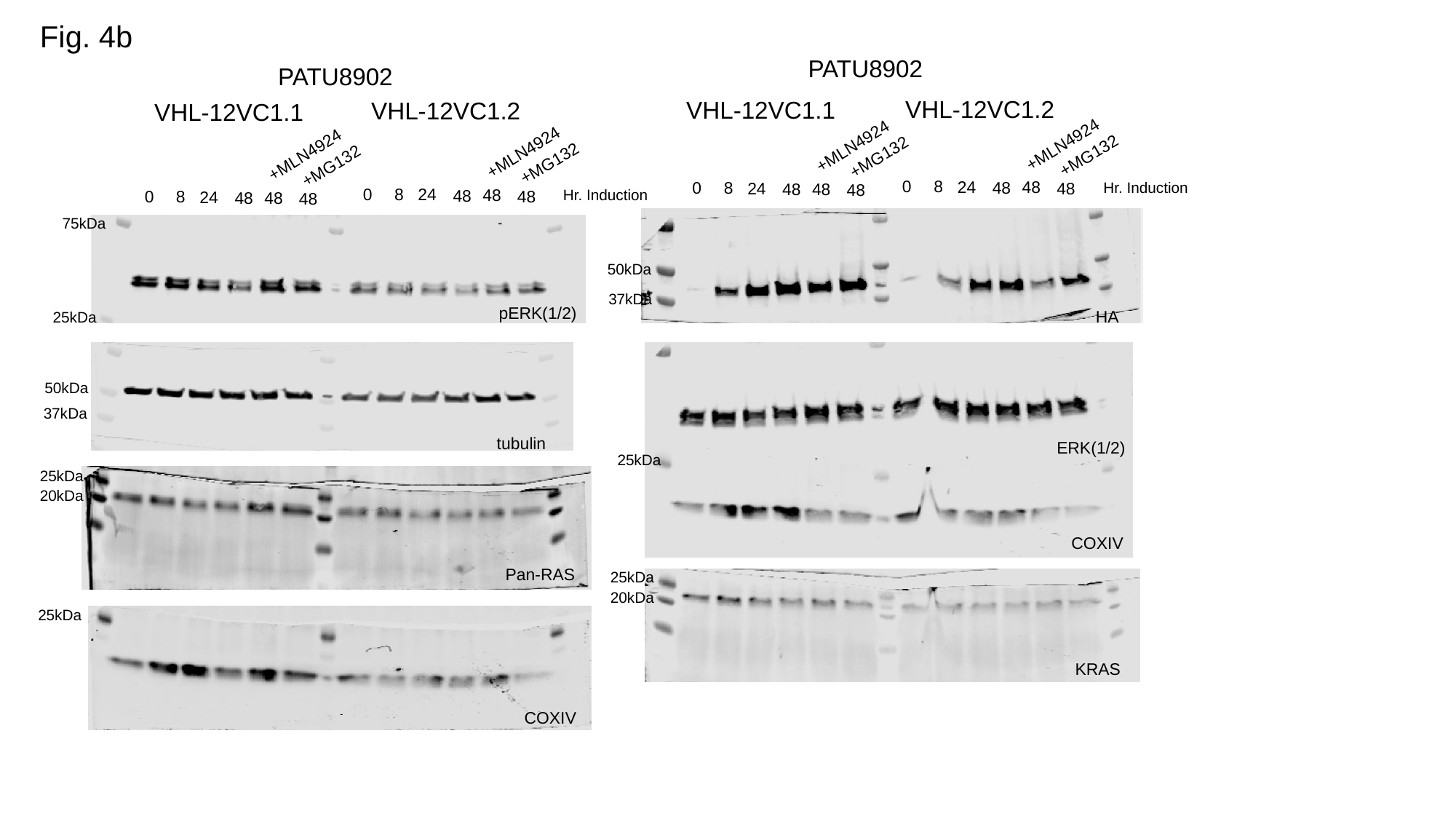

Fig. 4b
PATU8902
PATU8902
VHL-12VC1.2
VHL-12VC1.1
VHL-12VC1.2
VHL-12VC1.1
+MLN4924
+MLN4924
+MLN4924
+MLN4924
+MG132
+MG132
+MG132
+MG132
0
8
24
48
48
0
8
24
48
Hr. Induction
48
48
48
0
8
24
48
48
Hr. Induction
48
0
8
24
48
48
48
75kDa
50kDa
37kDa
pERK(1/2)
HA
25kDa
50kDa
37kDa
tubulin
ERK(1/2)
25kDa
25kDa
20kDa
COXIV
Pan-RAS
25kDa
20kDa
25kDa
KRAS
COXIV

## Slide 7
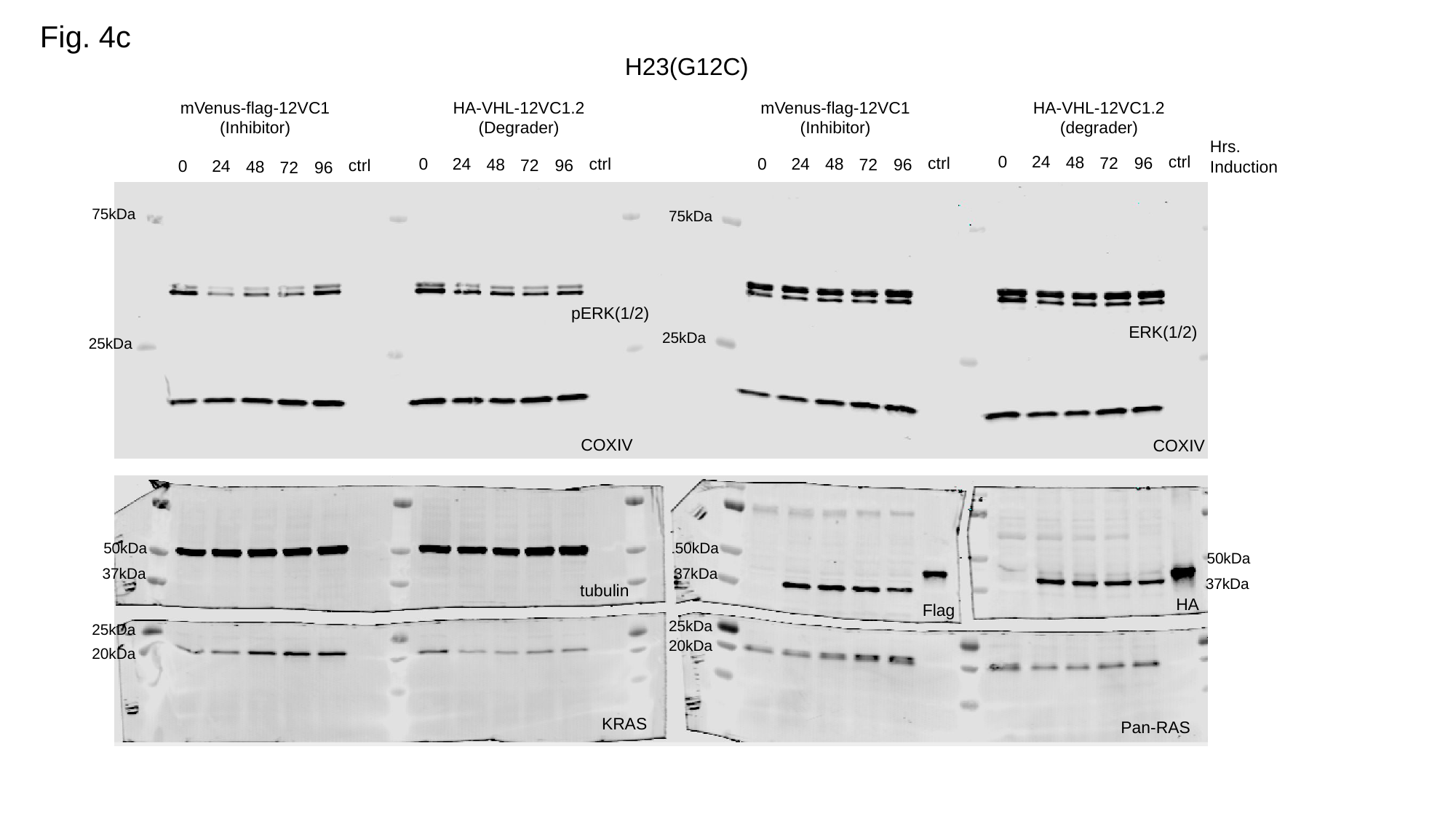

Fig. 4c
H23(G12C)
mVenus-flag-12VC1
(Inhibitor)
HA-VHL-12VC1.2
(Degrader)
mVenus-flag-12VC1
(Inhibitor)
HA-VHL-12VC1.2
(degrader)
Hrs.
Induction
ctrl
0
24
48
ctrl
72
96
0
24
ctrl
48
0
24
48
72
96
ctrl
72
96
0
24
48
72
96
75kDa
75kDa
pERK(1/2)
ERK(1/2)
25kDa
25kDa
COXIV
COXIV
50kDa
50kDa
50kDa
37kDa
37kDa
37kDa
tubulin
HA
Flag
25kDa
25kDa
20kDa
20kDa
KRAS
Pan-RAS

## Slide 8
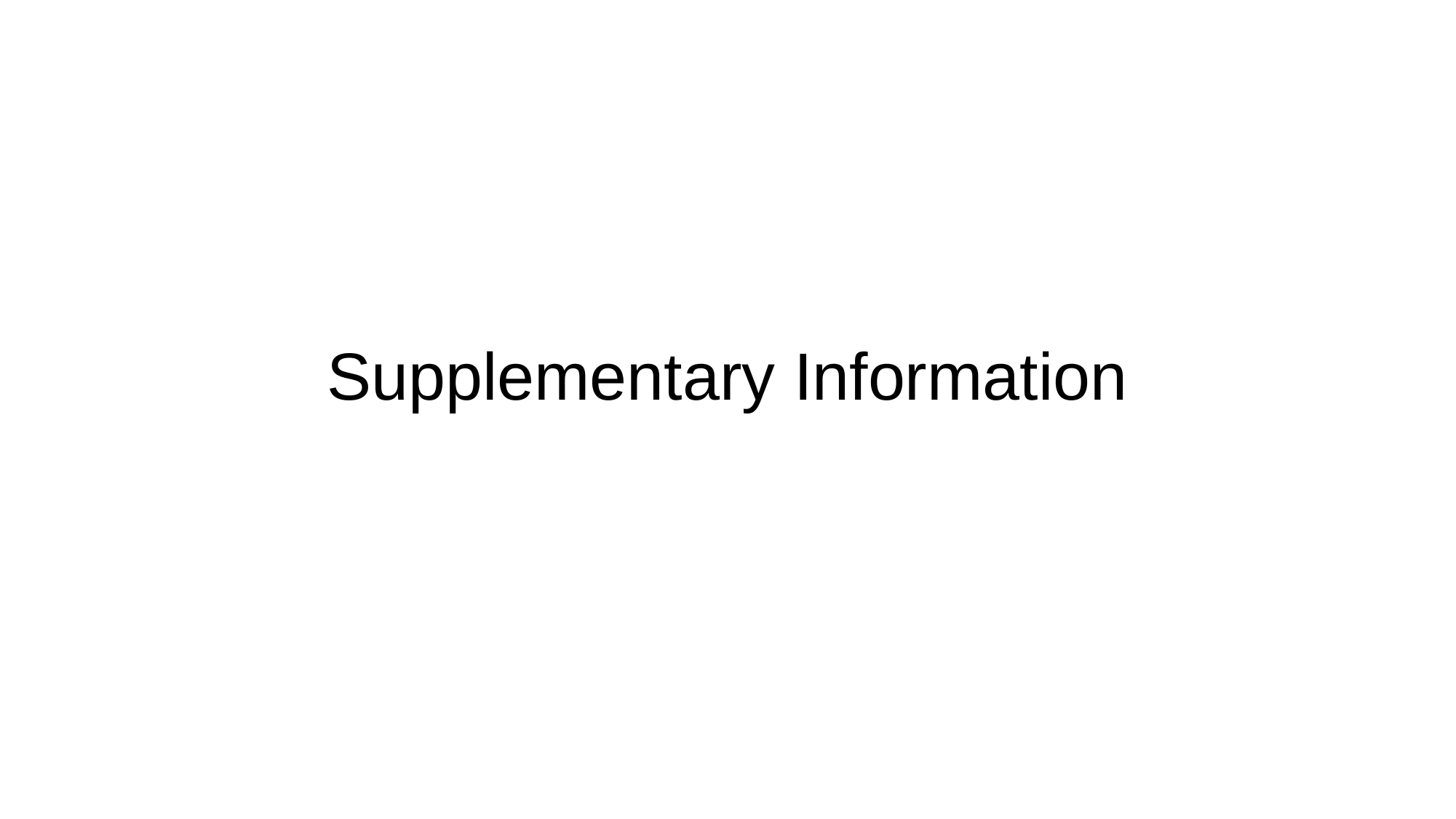

# Supplementary Information

## Slide 9
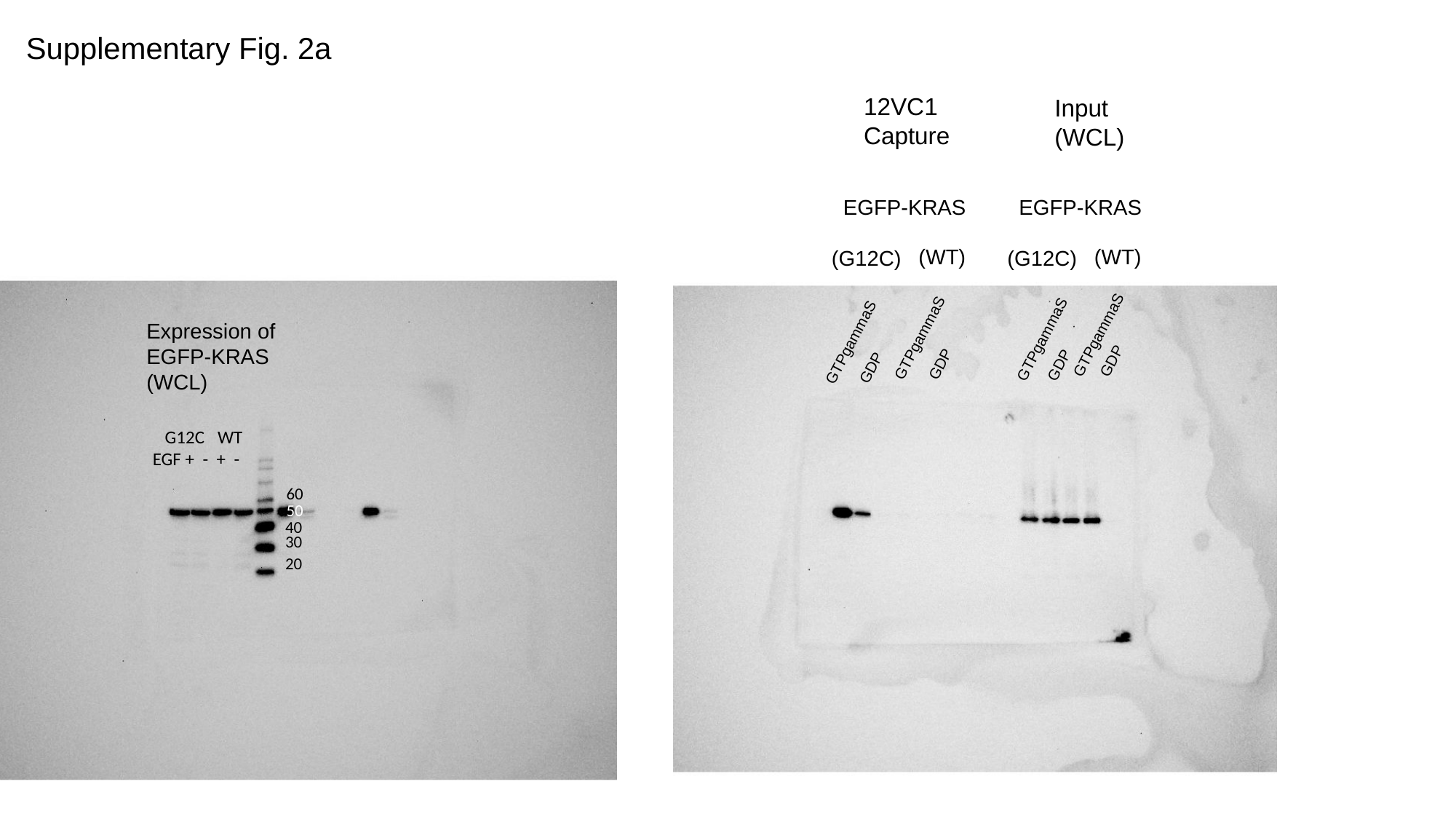

Supplementary Fig. 2a
12VC1Capture
Input
(WCL)
EGFP-KRAS
EGFP-KRAS
(WT)
(WT)
(G12C)
(G12C)
GTPgammaS
GDP
GTPgammaS
GDP
GTPgammaS
GDP
GDP
GTPgammaS
Expression of EGFP-KRAS
(WCL)
G12C
WT
EGF + - + -
60
50
40
30
20

## Slide 10
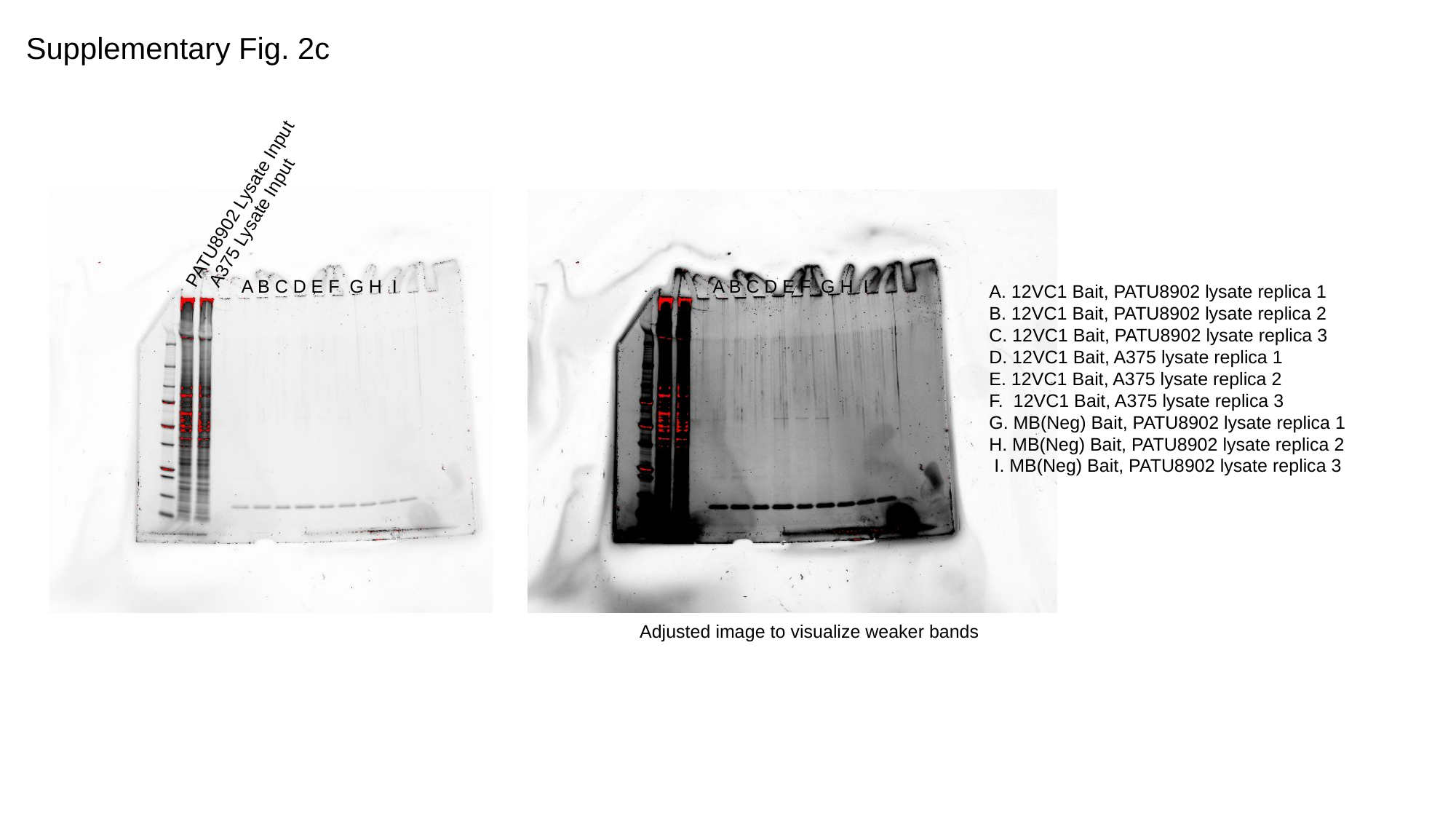

Supplementary Fig. 2c
A375 Lysate Input
PATU8902 Lysate Input
A B C D E F G H I
A B C D E F G H I
A. 12VC1 Bait, PATU8902 lysate replica 1
B. 12VC1 Bait, PATU8902 lysate replica 2
C. 12VC1 Bait, PATU8902 lysate replica 3
D. 12VC1 Bait, A375 lysate replica 1
E. 12VC1 Bait, A375 lysate replica 2
F. 12VC1 Bait, A375 lysate replica 3
G. MB(Neg) Bait, PATU8902 lysate replica 1
H. MB(Neg) Bait, PATU8902 lysate replica 2
 I. MB(Neg) Bait, PATU8902 lysate replica 3
Adjusted image to visualize weaker bands

## Slide 11
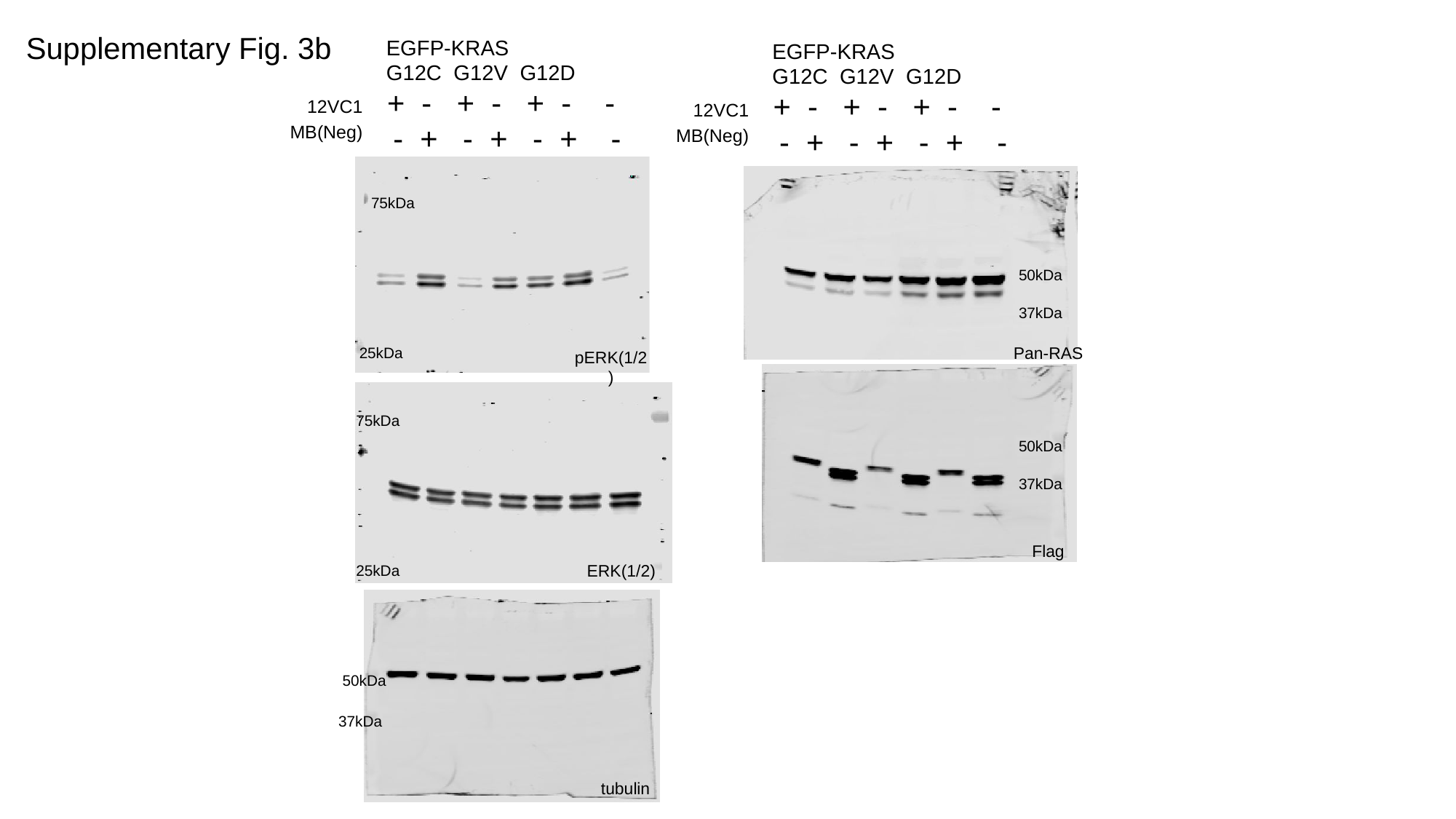

Supplementary Fig. 3b
EGFP-KRAS
G12C G12V G12D
EGFP-KRAS
G12C G12V G12D
+ - + - + - -
+ - + - + - -
12VC1
12VC1
- + - + - + -
MB(Neg)
- + - + - + -
MB(Neg)
75kDa
50kDa
37kDa
Pan-RAS
25kDa
pERK(1/2)
75kDa
50kDa
37kDa
Flag
ERK(1/2)
25kDa
50kDa
37kDa
tubulin

## Slide 12
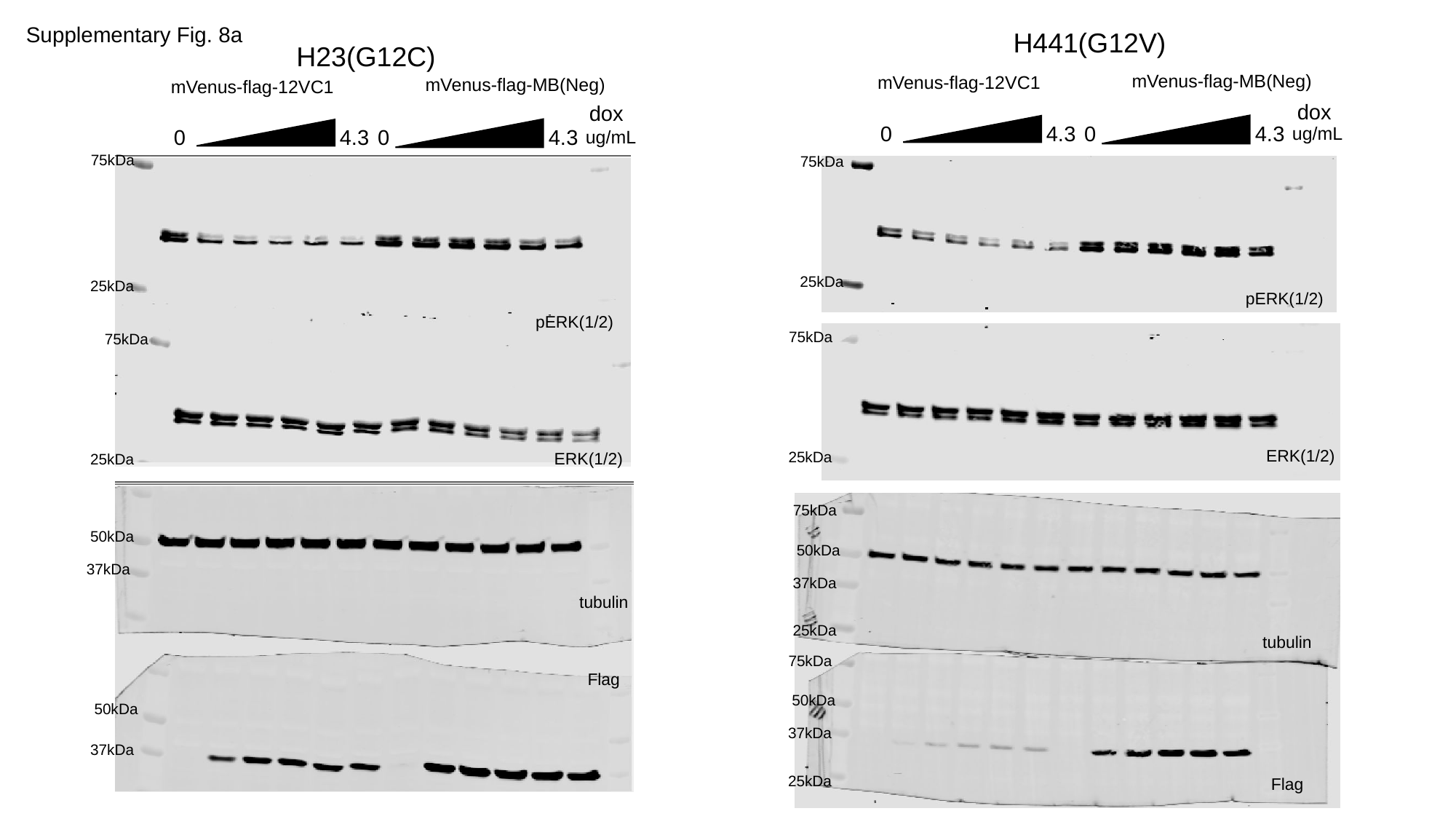

Supplementary Fig. 8a
H441(G12V)
H23(G12C)
mVenus-flag-MB(Neg)
mVenus-flag-12VC1
mVenus-flag-MB(Neg)
mVenus-flag-12VC1
dox
dox
0
4.3
0
4.3
ug/mL
0
4.3
0
4.3
ug/mL
75kDa
75kDa
25kDa
25kDa
pERK(1/2)
pERK(1/2)
75kDa
75kDa
ERK(1/2)
25kDa
ERK(1/2)
25kDa
75kDa
50kDa
50kDa
37kDa
37kDa
tubulin
25kDa
tubulin
75kDa
Flag
50kDa
50kDa
37kDa
37kDa
25kDa
Flag

## Slide 13
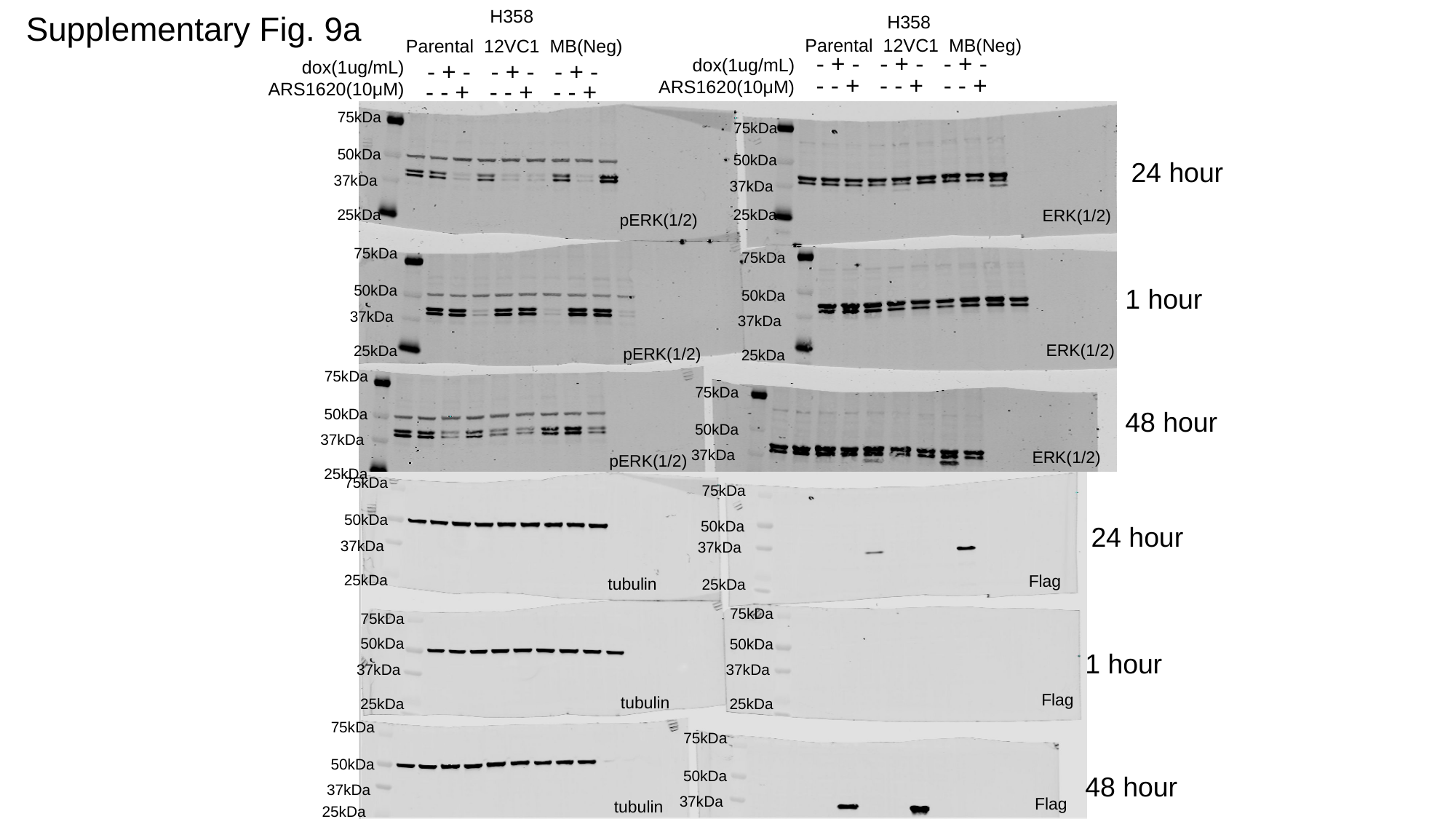

H358
H358
Supplementary Fig. 9a
Parental 12VC1 MB(Neg)
Parental 12VC1 MB(Neg)
- + - - + - - + -
dox(1ug/mL)
ARS1620(10μM)
dox(1ug/mL)
ARS1620(10μM)
- + - - + - - + -
- - + - - + - - +
- - + - - + - - +
75kDa
75kDa
50kDa
50kDa
24 hour
37kDa
37kDa
25kDa
25kDa
ERK(1/2)
pERK(1/2)
75kDa
75kDa
50kDa
1 hour
50kDa
37kDa
37kDa
ERK(1/2)
25kDa
pERK(1/2)
25kDa
75kDa
75kDa
50kDa
48 hour
50kDa
37kDa
37kDa
ERK(1/2)
pERK(1/2)
25kDa
75kDa
75kDa
50kDa
50kDa
24 hour
37kDa
37kDa
Flag
25kDa
tubulin
25kDa
75kDa
75kDa
50kDa
50kDa
1 hour
37kDa
37kDa
Flag
tubulin
25kDa
25kDa
75kDa
75kDa
50kDa
50kDa
48 hour
37kDa
37kDa
Flag
tubulin
25kDa

## Slide 14
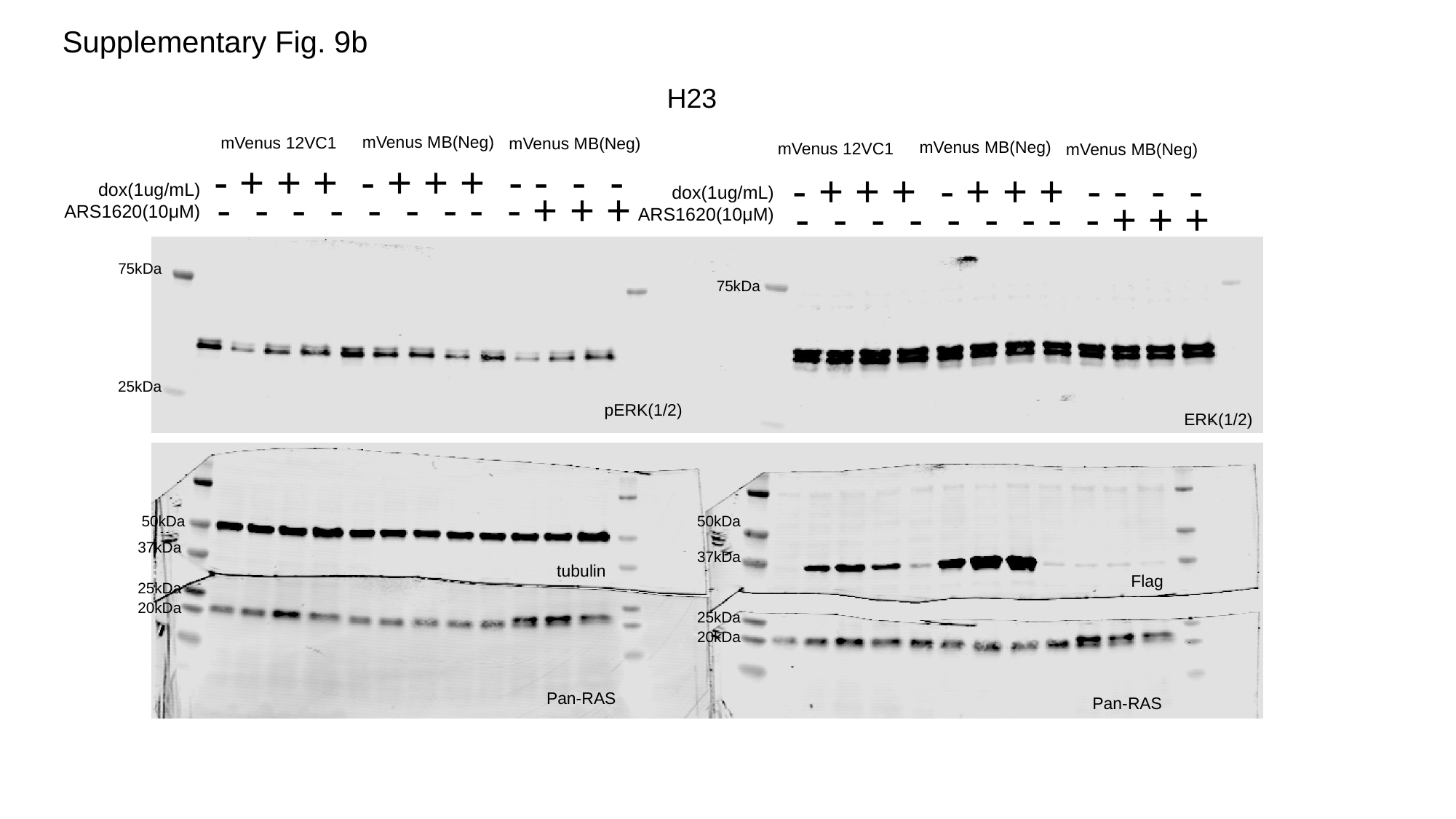

Supplementary Fig. 9b
H23
mVenus MB(Neg)
mVenus 12VC1
mVenus MB(Neg)
mVenus MB(Neg)
mVenus 12VC1
mVenus MB(Neg)
- + + + - + + + - - - -
- + + + - + + + - - - -
dox(1ug/mL)
ARS1620(10μM)
dox(1ug/mL)
ARS1620(10μM)
- - - - - - - - - + + +
- - - - - - - - - + + +
75kDa
75kDa
25kDa
pERK(1/2)
ERK(1/2)
50kDa
50kDa
37kDa
37kDa
tubulin
Flag
25kDa
20kDa
25kDa
20kDa
Pan-RAS
Pan-RAS

## Slide 15
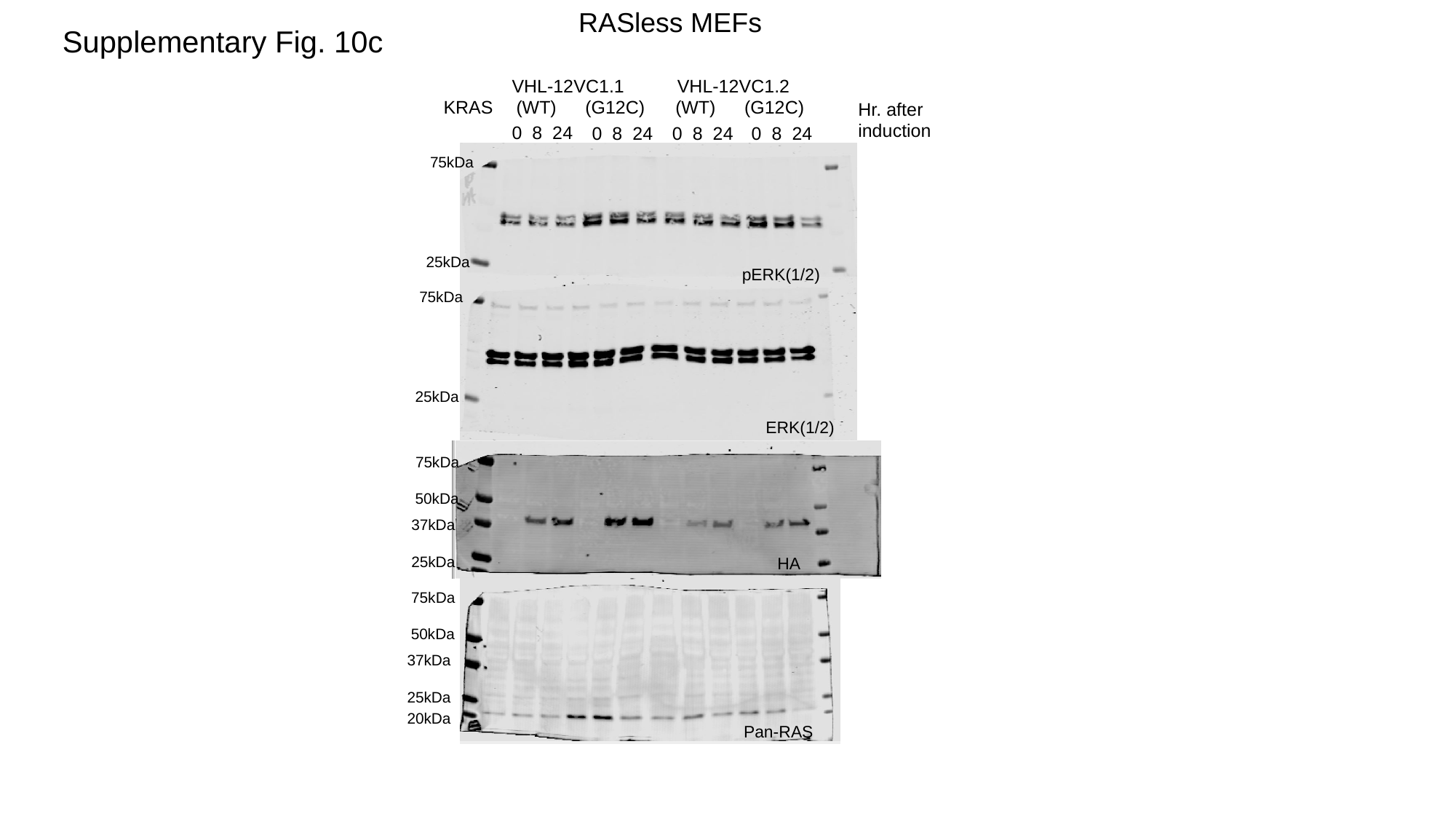

RASless MEFs
Supplementary Fig. 10c
VHL-12VC1.1
VHL-12VC1.2
KRAS
(G12C)
(G12C)
(WT)
(WT)
Hr. after induction
0 8 24
0 8 24
0 8 24
0 8 24
75kDa
25kDa
pERK(1/2)
75kDa
25kDa
ERK(1/2)
75kDa
50kDa
37kDa
25kDa
HA
75kDa
50kDa
37kDa
25kDa
20kDa
Pan-RAS

## Slide 16
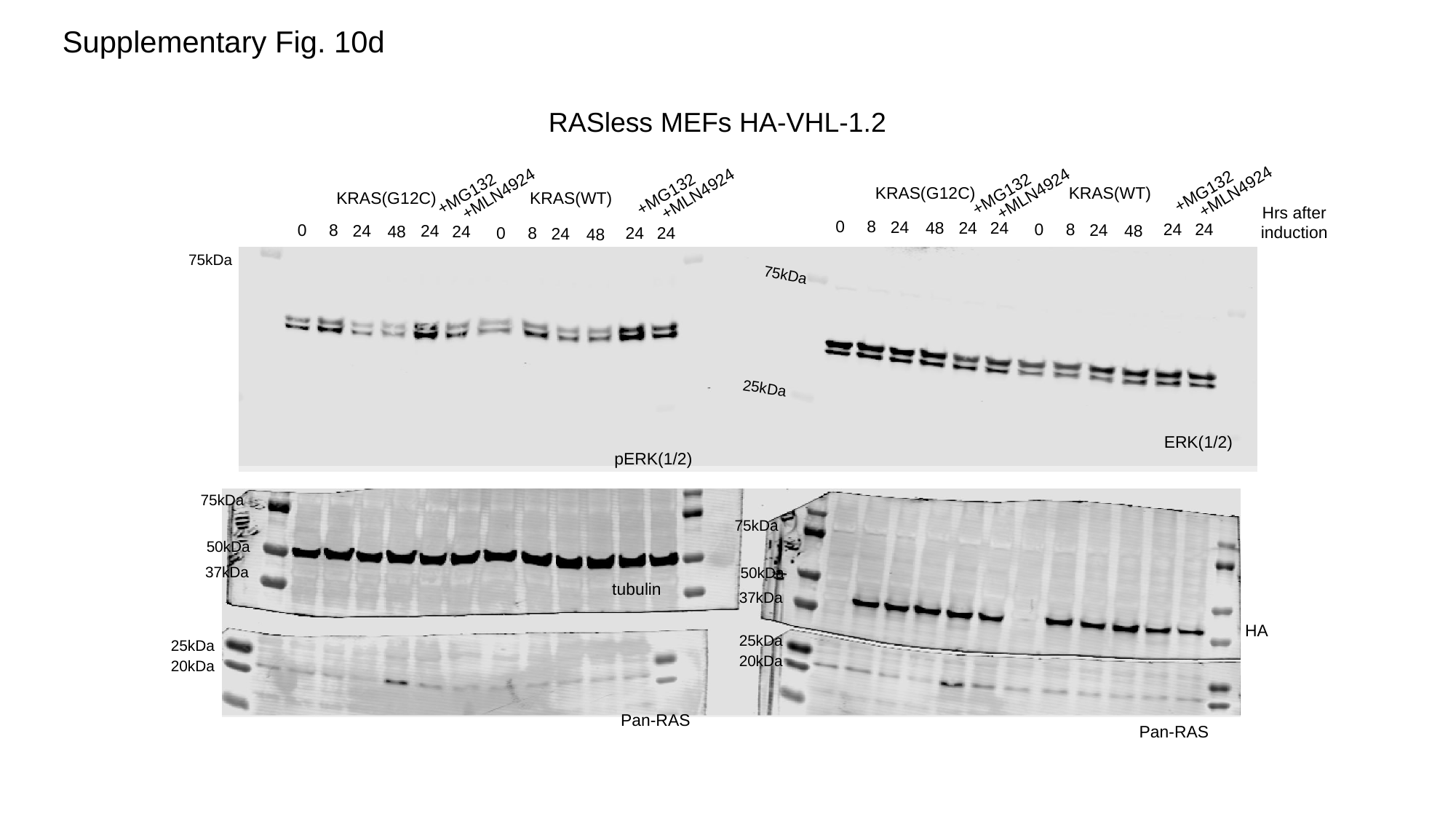

Supplementary Fig. 10d
RASless MEFs HA-VHL-1.2
+MG132
+MLN4924
KRAS(G12C)
KRAS(WT)
+MG132
+MLN4924
+MG132
+MLN4924
+MG132
+MLN4924
KRAS(G12C)
KRAS(WT)
Hrs after induction
0
8
24
24
24
48
24
0
8
24
24
0
8
48
24
24
24
48
24
0
8
24
24
48
75kDa
75kDa
25kDa
ERK(1/2)
pERK(1/2)
75kDa
75kDa
50kDa
37kDa
50kDa
tubulin
37kDa
HA
25kDa
25kDa
20kDa
20kDa
Pan-RAS
Pan-RAS

## Slide 17
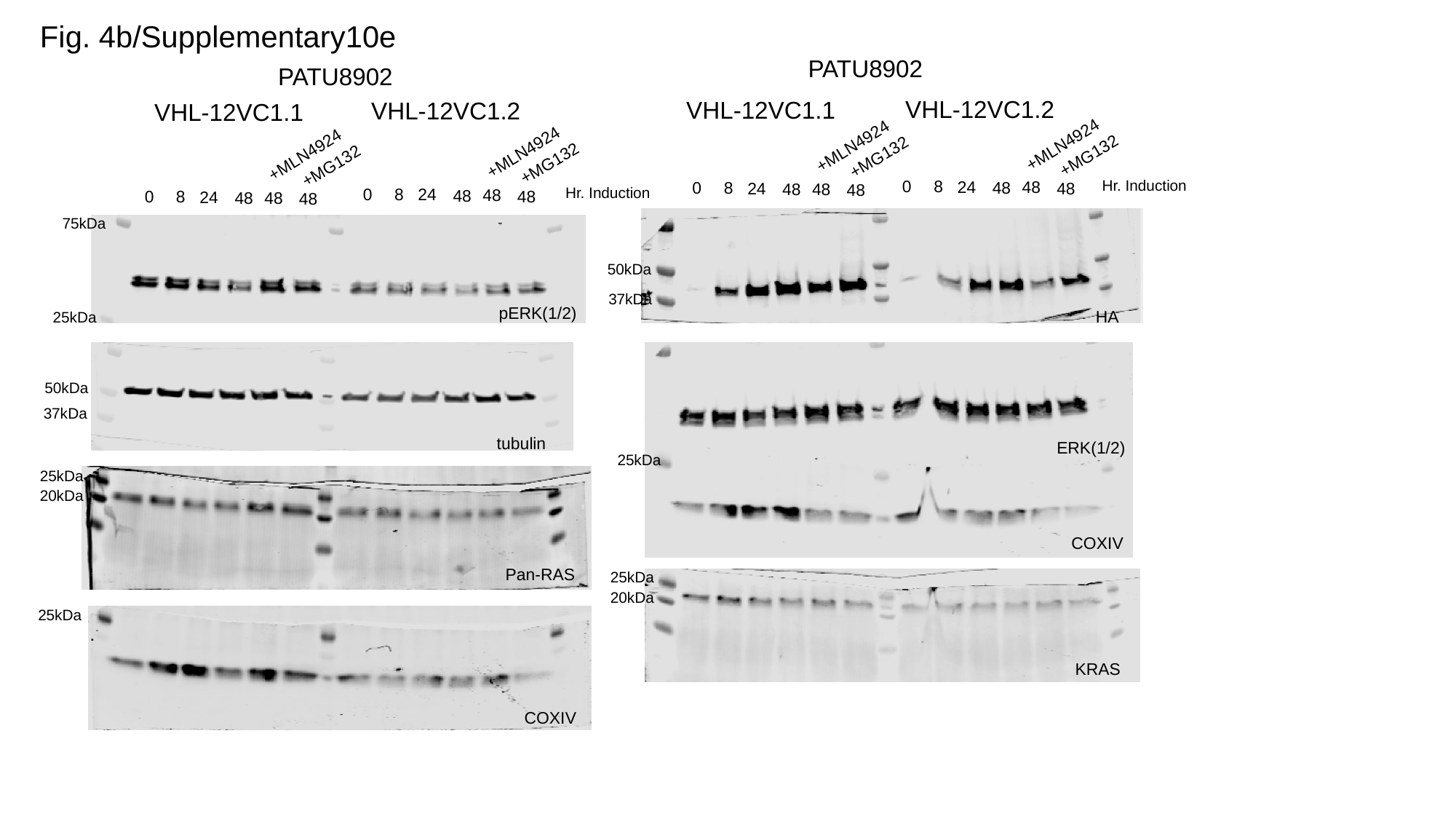

Fig. 4b/Supplementary10e
PATU8902
PATU8902
VHL-12VC1.2
VHL-12VC1.1
VHL-12VC1.2
VHL-12VC1.1
+MLN4924
+MLN4924
+MLN4924
+MLN4924
+MG132
+MG132
+MG132
+MG132
Hr. Induction
0
8
24
48
48
0
8
24
48
48
48
48
Hr. Induction
0
8
24
48
48
48
0
8
24
48
48
48
75kDa
50kDa
37kDa
pERK(1/2)
HA
25kDa
50kDa
37kDa
tubulin
ERK(1/2)
25kDa
25kDa
20kDa
COXIV
Pan-RAS
25kDa
20kDa
25kDa
KRAS
COXIV

## Slide 18
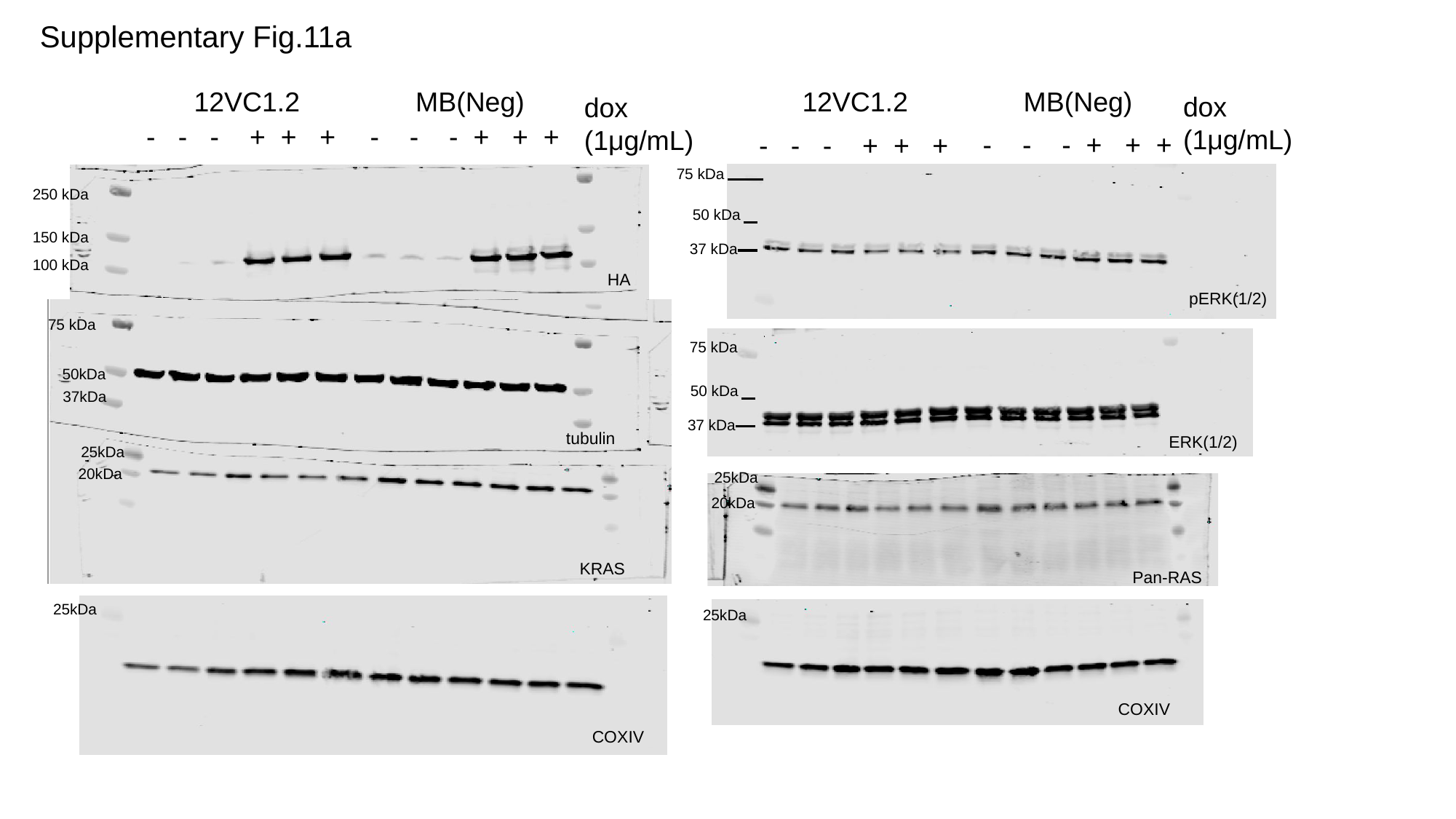

Supplementary Fig.11a
12VC1.2
MB(Neg)
12VC1.2
MB(Neg)
dox (1μg/mL)
dox (1μg/mL)
- - - + + +
- - - + + +
- - - + + +
- - - + + +
75 kDa
250 kDa
150 kDa
100 kDa
75 kDa
50kDa
37kDa
25kDa
20kDa
50 kDa
37 kDa
HA
pERK(1/2)
75 kDa
50 kDa
37 kDa
tubulin
ERK(1/2)
25kDa
20kDa
KRAS
Pan-RAS
25kDa
25kDa
COXIV
COXIV

## Slide 19
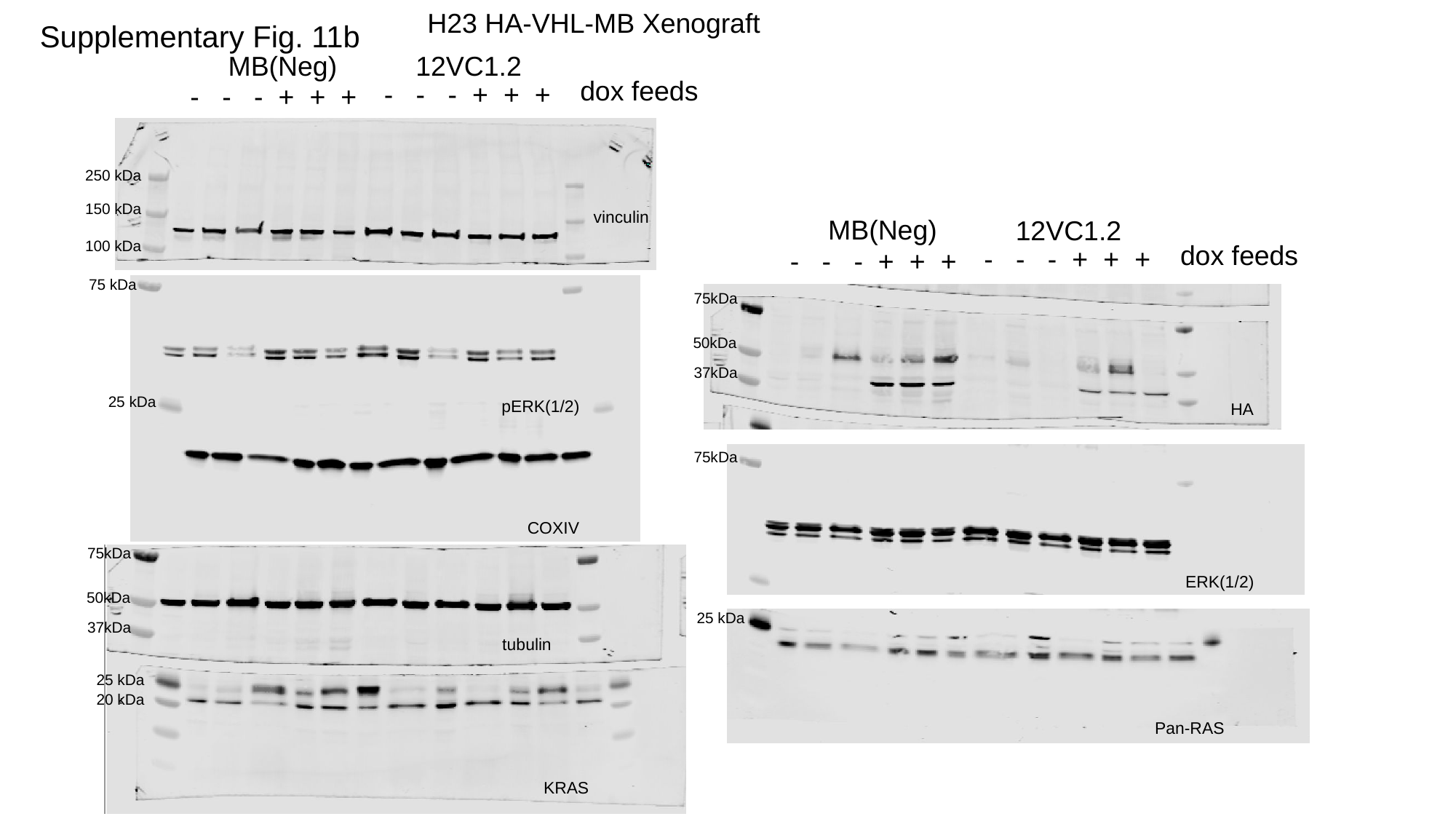

H23 HA-VHL-MB Xenograft
Supplementary Fig. 11b
MB(Neg)
12VC1.2
dox feeds
- - - + + +
- - - + + +
250 kDa
150 kDa
100 kDa
75 kDa
25 kDa
vinculin
MB(Neg)
12VC1.2
dox feeds
- - - + + +
- - - + + +
75kDa
50kDa
37kDa
pERK(1/2)
HA
75kDa
COXIV
75kDa
50kDa
37kDa
25 kDa
20 kDa
ERK(1/2)
25 kDa
tubulin
Pan-RAS
KRAS
